# Lifting the veil: probing altered visual perception in derealization

**DOI:** 10.1093/nc/niaf045

**Published:** 2025-11-23

**Authors:** Anikó Kusztor, Nirmitee Mulay, Makiko Yamada, Jakob Hohwy, Naotsugu Tsuchiya

**Affiliations:** School of Psychological Sciences, Monash University, 35 Rainforest Walk, Clayton Campus, Clayton, VIC 3800, Australia; Department of Biological Sciences, Indian Institute of Science Education and Research Kolkata (IISER Kolkata), Campus Rd, Mohanpur 741246, West Bengal, India; Basque Center on Cognition, Brain and Language, 20009 San Sebastian, Gipuzkoa, Spain; University of the Basque Country- UPV/EHU, 20018 San Sebastian, Gipuzkoa, Spain; Institute for Quantum Medical Science, National Institutes for Quantum Science and Technology, 4-9-1 Anagawa, Inage-ku, Chiba-shi, Chiba 263-8555, Japan; Institute for Quantum Life Science, National Institutes for Quantum Science and Technology, 4-9-1 Anagawa, Inage-ku, Chiba-shi, Chiba 263-8555, Japan; Department of Quantum Life Science, Graduate School of Science, Chiba University, Chiba, Japan; Graduate School of Medicine, Tohoku University, Miyagi, Japan; Monash Centre for Consciousness & Contemplative Studies, Monash University, 4th Floor/29 Ancora Imparo Way, Clayton, VIC 3800, Australia; School of Psychological Sciences, Monash University, 35 Rainforest Walk, Clayton Campus, Clayton, VIC 3800, Australia; Turner Institute for Brain and Mental Health & School of Psychological Sciences, Faculty of Medicine, Nursing, and Health Sciences, Monash University,18 Innovation Walk, Clayton, VIC 3800, Australia; Center for Information and Neural Networks (CiNet), National Institute of Information and Communications Technology (NICT), 1-4 Yamadaoka, Suita, Osaka 565-0871, Japan; Laboratory of Qualia Structure, ATR Computational Neuroscience Laboratories, 2-2-2 Hikaridai, Seika-cho, Soraku-gun, Kyoto Prefecture 619-0288, Japan

**Keywords:** Derealisation, depersonalisation, vividness, perception

## Abstract

During an acute episode of depersonalization/derealization (DP/DR), people report a complex and idiosyncratic change in their perceptual experience. Specifically, derealisation describes the experience of detachment from the external world and altered visual perception in which the surroundings look faded, foggy, or dream-like. Whilst some have argued that there may not be genuine perceptual changes in derealization, this proposal is yet to be tested empirically. Thus, we set out to investigate the potential perceptual changes in derealization. In this Registered Report, we conducted two online experiments to reveal the impact of DP/DR symptoms measured *via* the state version of the Cambridge Depersonalisation Scale (CDS) on how people evaluate (Experiment 1, *N* = 200, CDS-state mean: 32.43 ± 29.94 SD) and adjust (Experiment 2, *N* = 125, CDS-state mean: 29.38 ± 30.47 SD) naturalistic scene images with different levels of saturation and contrast. Participants were asked to rate how real the presented images look compared to their everyday experience (in Experiment 1) and to adjust the contrast or saturation level of images to match their everyday visual experience (in Experiment 2). We tested the effect of CDS-state scores on these subjective ratings *via* model comparison with Bayes Factors. In both experiments, we found strong evidence supporting the null models, suggesting that DP/DR symptoms did not affect realness ratings or vividness adjustments. These results provide empirical support for theories suggesting that self-reported altered vividness experience in derealization does not reflect genuine perceptual changes, instead they signify the (meta-)cognitive interpretation of these experiences. We discuss pros and cons of the current research practices when assessing derealization and highlight key avenues for the future investigation.

## Introduction

Most of us move through our day-to-day life with a sense that our experiences reflect reality without any doubt. Some may even find it hard to answer when asked if their surroundings look real to them because they have never questioned it before. Under some circumstances, however, this ongoing sense of reality becomes compromised. Conditions such as acute stress (Baker et al. 2003; Hunter et al. 2017), being under the influence of psychedelic substances (Mathew et al. 1999; Medford et al. 2003; Niciu et al. 2018), sleep deprivation (Waters et al. 2018), or meditation (Castillo 1990) can all profoundly change whether we experience ourselves and the external world as part of reality.

Depersonalization and derealization experiences are prevalent in both clinical and general populations (Hunter et al. 2004), but many of their experiential characteristics have so far remained unclear. From a diagnostic point of view, depersonalization describes the experience of the unreality of one’s self, and derealization is defined as experiencing detachment or unreality of the surrounding environment (American Psychiatric Association 2013). Although these experiences frequently occur together, they can be distinguished in terms of subjective experiences as well as physiological and neural underpinnings (Sierra et al. 2002; Dutt and Grover 2009; Ratnam et al. 2016; Dewe et al. 2018). Even so, most psychological inventories measure depersonalization (DP) and derealization (DR) together. In this article, therefore, we will use the term DP/DR when we cannot discuss derealization separately. Nonetheless, the primary target of this research is derealization.

According to subjective reports and diagnostic guides, derealization involves visual experiences with altered colour intensity, fogginess, flatness, and other perceptual distortions such as changes in distances and size (Sierra and Berrios 2000; Hunter et al. 2003; Sar et al. 2017; Ciaunica et al. 2022b). These perceptual alterations can be divided the following way: metamorphopsia (changes of shapes and colours), micropsia/macropsia (changes in size), and teleopsia (objects look more distant) (Lipsanen et al. 1999). Besides these specific distortions, phenomenological descriptions often emphasize decreased vividness or dullness of the external world (Pienkos and Sass 2022). When discussing vividness, it is important to note that in some instances, vividness refers to colour intensity only (e.g. Medford 2012; Yokokawa et al. 2018). However, vividness is perhaps better conceptualized as a subjective quality that is the combination of intensity (i.e. apparent saturation, contrast, and brightness), specificity (i.e. blurriness and detailedness), and stability (i.e. length of presence in time) (Fazekas et al. 2020). In addition, terms like dream-likeness, artificialness, or the phrase ‘the world looks as if…’ are often used to explain how the world looks unreal (Dutt and Grover 2009; Hunter et al. 2017). Thus, it is unclear to what extent specific visual distortions can contribute to the overall experiences of unrealness.

Approaching this question from a theoretical angle, several recent papers put forward arguments in favour of conceptualizing derealization independently from perceptual distortions (Dokic and Martin 2017; Deroy and Rappe 2022; Gładziejewski 2022; Suzuki et al. 2023). According to Dokic and Martin (2017), derealization arises from a disruption in the ‘sense of reality’, which they described as a metacognitive feeling that accompanies experiences regardless of the sensory input. Others posited that it is a specific perceptual component that is diminished in derealization (Deroy and Rappe 2022; Gładziejewski 2022; Minden Ribeiro 2022; Suzuki et al. 2023). For example, Gładziejewski (2022) argues that the visual experience feels different in derealization because of diminished connectedness with the external world due to altered interoceptive processes. Suzuki et al. (2023) take a similar position, but they acknowledge that the vividness level of visual content can possibly contribute to derealization. Thus, theoretical proposals range from those that emphasize more cognitive processes to those emphasizing more perceptual processes that underlie derealization.

Currently, it is unclear whether realness is related to visual perceptual properties such as vividness. To address this issue, Yokokawa et al. (2018) investigated the experience of unreality and its association with perceived fadedness. They have found that healthy participants rated images with mid-level saturation as less real when the preceding image was highly saturated as opposed to weakly saturated. This apparent fadedness was associated with activity in the dorsolateral prefrontal and parietal cortex as well as striatal D2 receptor availability. These findings suggest that the subjective experience of colour saturation contributes to the perceptual judgements of what constitutes as real. Yet, the reason for this association remains unknown.

A clue to the link between realness and vividness might be that most people report experiencing internally generated percepts, such as mental imagery or dreams, as less vivid (Dawes et al. 2020; Dance et al. 2022). Under these ‘normatively unreal’ situations, people know that lower levels of vividness are associated with unreality. This logic may be invoked, either implicitly or explicitly, when people report unrealness in the instances when their perception becomes less vivid for some reason. Alternatively, this association might be invoked in post-perceptual reports. In line with this notion, people tend to report decreased colour saturation and loss of background details when asked to identify the visual characteristics of their dream upon awakening (e.g. Rechtschaffen and Buchignani 1992; for critique see: Schwitzgebel et al. 2006). Possibly, experiencing low vividness may also give rise to the sense of unrealness.

**Figure 1:**
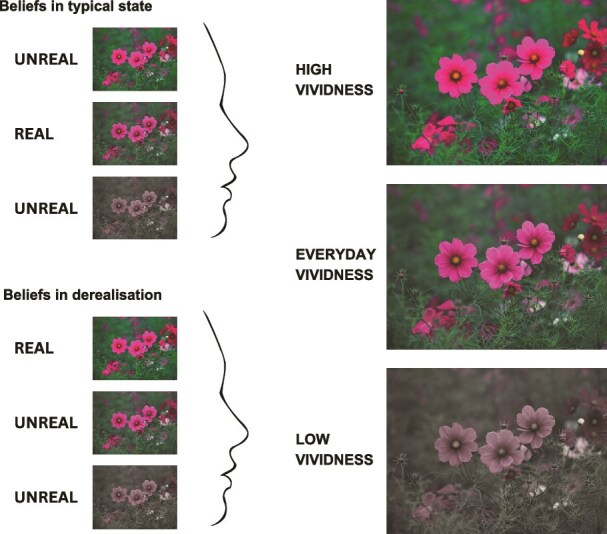
In derealization, individuals report that their visual experience is distinct from what they believe their environment usually looks in terms of perceptual features like vividness. We propose that by showing images with vividness levels that exceed vividness seen in the day-to-day experiences of most people, we can test how people differ in their beliefs about realness on natural images that are manipulated over a range of luminance contrast or colour saturation. A possible scenario is that a person without derealization would accept images with everyday vividness levels as close to reality, while a person experiencing derealization would consider images with everyday vividness levels as falling short of reality.

Here, we gauge the impact of different levels of vividness on what people perceive as real. We operationally define perceived realness as the degree of match between what people see and their internal model of ‘real’ experiences, i.e. what they consider normal in their everyday life. To invoke the comparison between the visual stimuli and the participants’ internal model of reality, we instruct the participants to use their day-to-day experiences as reference. Since people experiencing derealization tend to report altered visual experiences, we assume that referencing everyday experiences will prompt participants to make a comparison between the presented stimuli and their beliefs about reality. Critically, we will manipulate our experimental stimuli to encompass a wide range of vividness from unnaturally low to unnaturally high levels (see Fig. 1). Thus, we can systematically assess if the measured realness at particular vividness levels is associated with the experience of derealization.

In this registered report, we propose to conduct two sets of online psychophysics experiments in conjunction with self-reported assessments of DP/DR symptoms. In the first set of experiments, we will examine the impact of different image features contributing to vividness (i.e. saturation levels, contrast levels) on subjective judgements about how real an image looks compared to participants’ day-to-day experiences. To do this, we have selectively modified the saturation and contrast levels of naturalistic images in both directions. Following the findings by Yokokawa et al. (2018), we expect that less vivid images (i.e. lower saturation/contrast) will be rated as less real compared to more vivid images.

**Figure 2:**
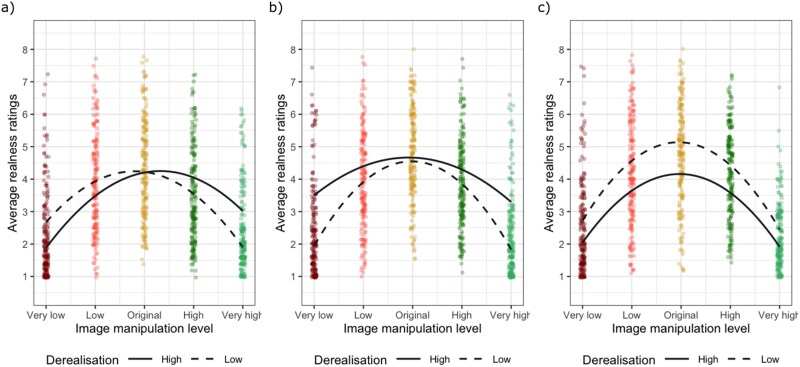
These figures show simulated data demonstrating examples for our proposed interpretations if H1 is supported. In order to simplify this visualization, we opted to use a quadratic polynomial term for modelling the image manipulation level based on the pilot data we have collected and modified only a single coefficient in the regression model for each example. We have simulated data from 200 participants. The dots represent average responses across participants.

Our main hypothesis (H1) is that ongoing DP/DR experiences will have an association with the realness ratings. If confirmed, we propose the following interpretations and show their visual illustrations using simulated data in Fig. 2.

We may find that people with more severe ongoing DP/DR experiences will judge low and normal levels of saturation as less real compared to people with less DP/DR experiences whilst judging high levels of saturation as more real (Fig. 2a). This finding would support the view that a perceptual shift occurs in experienced vividness in derealization.

Alternatively, it is possible that more severe DP/DR will result in lower realness ratings for the modified images selectively but not for the original images (Fig. 2b). This finding would support the notion of a possible hypersensitivity in the perceptual processing of individuals with derealization in line with some recent proposals (Ciaunica et al. 2022a).

Finally, it is also possible severe DP/DR will lead to lower realness ratings across all manipulation levels (Fig. 2c). This finding would support the view that realness judgements in derealization are independent of apparent vividness, and they are informed by meta-cognitive processes rather than perceptual cues (e.g. Dokic and Martin 2017).

In the second set of experiments, we aim to measure how consistently people can adjust images to fit their day-to-day experiences with regard to saturation and contrast levels. Rivera-Aparicio and colleagues recently demonstrated that people consistently overestimate the clarity and colourfulness of previously seen images of natural scenes (Rivera-Aparicio et al. 2021). They have coined the term ‘vividness extension’ to describe this phenomenon.

**Figure 3:**
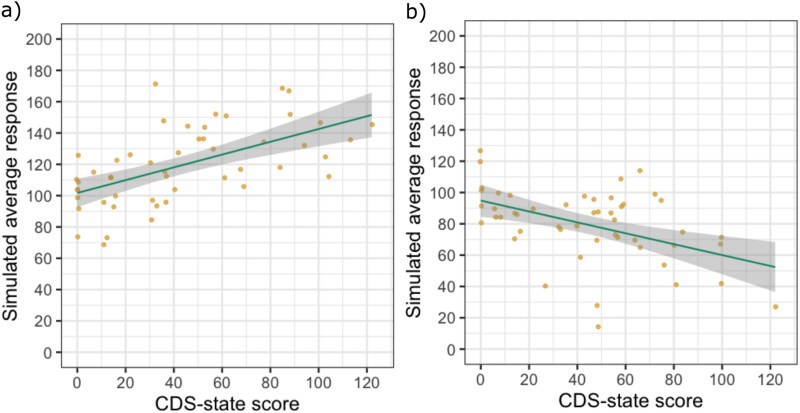
We have again simulated two example datasets demonstrating the proposed interpretations if H2 is supported. The dots represent average responses of 50 simulated participants.

Adapting their experiment, we hypothesize that DP/DR experiences will influence the levels of vividness (i.e. saturation and contrast) people select in the adjusted images (H2). We again describe our proposed interpretations below and present the corresponding visual illustrations in Fig. 3.

If DP/DR experiences show an association with the vividness of the adjusted images, we may find people with more severe DP/DR produce more vivid images when asked to match their day-to-day experiences (Fig. 3a). This could happen because individuals experiencing derealization may overestimate how vivid their experiences usually are.

Alternatively, severe DP/DR experiences may lead to producing less vivid images (Fig. 3b). This could indicate individuals experiencing derealization may be inclined to recreate their day-to-day experience with images matching the subjective reports of decreased vividness.

With these experiments, we aim to shed light on the relationship between the vividness of perceptual experiences and an altered sense of realness in derealization. To this end, we ask participants from a non-clinical population with varying severity of DP/DR experiences to evaluate for each presented image, with its particular level of vividness, if it corresponds to ‘what the world usually looks like’, i.e. their beliefs about realness. This means we aim to gauge participants’ mapping between vividness and realness. Notably, this is not meant to reveal changes in vividness perception *per se*; doing so would in effect be succumbing to the El Greco fallacy, which arises when asking participants to report perceptual attributes of a target stimulus using a reporting tool that invokes the same perceptual attribute (Firestone and Scholl 2014; see detailed discussion of this question in the supplementary materials). Instead, our goal is to test if certain image features contributing to vividness relate differently to beliefs about realness in people with unreal visual experiences. This approach allows us to provide empirical data on this much debated connection between vividness and realness.

One theoretical possibility is that all experiences in derealization are less vivid, including one’s internal model and beliefs of what the world usually looks like. In this case, the relationship between different levels of vividness could be maintained regardless of derealization severity. While we cannot refute this possibility, it seems unlikely. The reason for this is that people experiencing derealization spontaneously report deviations in their visual experience from what they consider as their usual ‘real-like’ experience, which they would not be able to do if the relationship between beliefs and visual experience was adapted in the way in question.

Thus, we find it reasonable to assume that these reports occur due to a mismatch between the current experience and the template of what the world usually looks like. Moreover, since our study targets a non-clinical population with most likely transient derealization, we think referring to the participants’ day-to-day experience is appropriate in this instance. Yet, we acknowledge that these assumptions may not generalize to all manifestations of derealization disorder.

## Methods

### Materials

#### Realness-rating task (Experiment 1)

We plan to conduct separate experiments with two types of image manipulation: saturation levels (Experiment 1a) and contrast levels (Experiment 1b). We have created and piloted this task using PsychoPy/Js (version 2021.1.4, RRID:SCR_006571) (Peirce et al. 2019). The stimuli consist of the same 100 naturalistic images with four distinct categories of image content: images depicting flowers (25) randomly selected from the stimulus set of Yokokawa et al. (2018), images showing inside (25) and outside (25) environments without visible people present selected form the Scene UNderstanding (SUN) database (Xiao et al. 2010), and images showing people in both outdoor and indoor settings (25) selected from the stimulus set used by Chuyin et al. (2022). All stimuli are available on the project’s online repository page.

**Figure 4:**
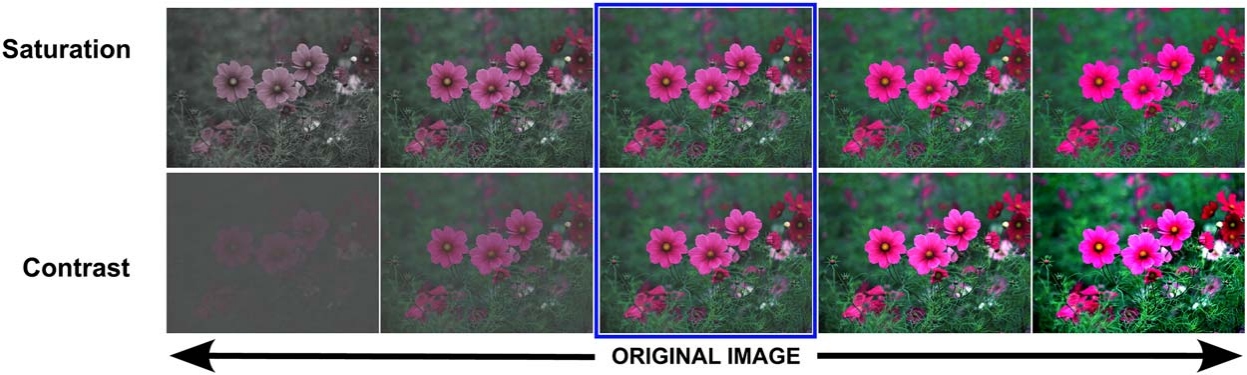
Examples of image manipulations for saturation and contrast levels showing the unmodified image as the original image and two progressively increasing and decreasing versions of the manipulated property.

We have modified specific properties of these images using the Image Enhance module from the Pillow library (version 8.2.0, RRID:SCR_023337) *via* Python Programming Language (RRID:SCR_008394). For each image, we have used the unmodified version of the images as well as two versions with high levels (high and very high) and two versions with low levels (low and very low) for a certain property (i.e. saturation, contrast) as shown on Fig. 4. Thus, for each experiment, we had five different versions of the 100 images (i.e. very low, low, original, high, very high).

We counterbalance the presentation of the different image manipulation versions within the experiments so that participants view 100 unique images with all manipulation levels equally represented but without seeing the same image more than once at different levels. Thus, each participant sees 20 images at each of the five manipulation levels adding up to 100 stimuli per participant.

**Figure 5:**
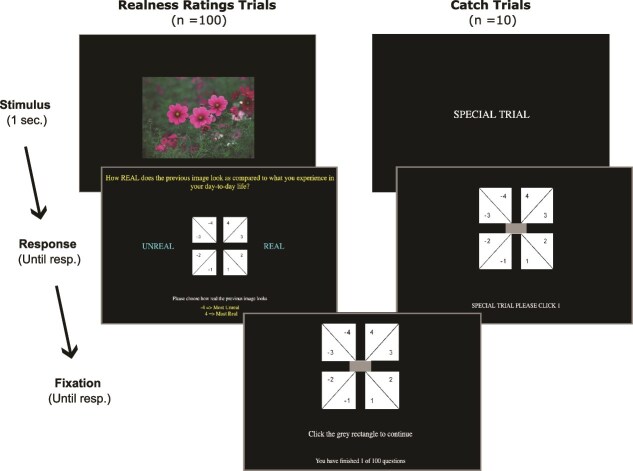
Illustration of realness rating task. The realness rating trials and catch trials were presented in a pseudo-randomized order.

The images are in the middle of the screen for 1 s in randomized order across participants. After each image, the participants see the response screen with an 8-point circular response scale (−4, −3, −2, −1, 1, 2, 3, 4); −4 being the most ‘Unreal’ and +4 being the most ‘Real’. The participant’s task is to evaluate how real the displayed image looks compared to what they usually tend to see in their day-to-day life. In addition, we include 10 randomized catch trials to identify inattentive responders, during which the participants see the text ‘Special trial’ in the middle of the screen for 1 s and then are asked to choose a specific number on the response scale (see Fig. 5). The experiment is hosted online on Pavlovia.org (RRID:SCR_023320).

#### Image adjustment task (Experiment 2)

For the image adjustment task, we will again focus on saturation (Experiment 2a) and contrast manipulation (Experiment 2b). The experiments have also been already piloted. We have created the task using jsPsych (version 7.2) (de Leeuw 2015). The task includes 40 different images presented twice. We have selected a subset of the images from Experiment 1 with 10 items from all four image content categories (i.e. 10 flowers, 10 inside environments, 10 outside environments, 10 images with people).

**Figure 6:**
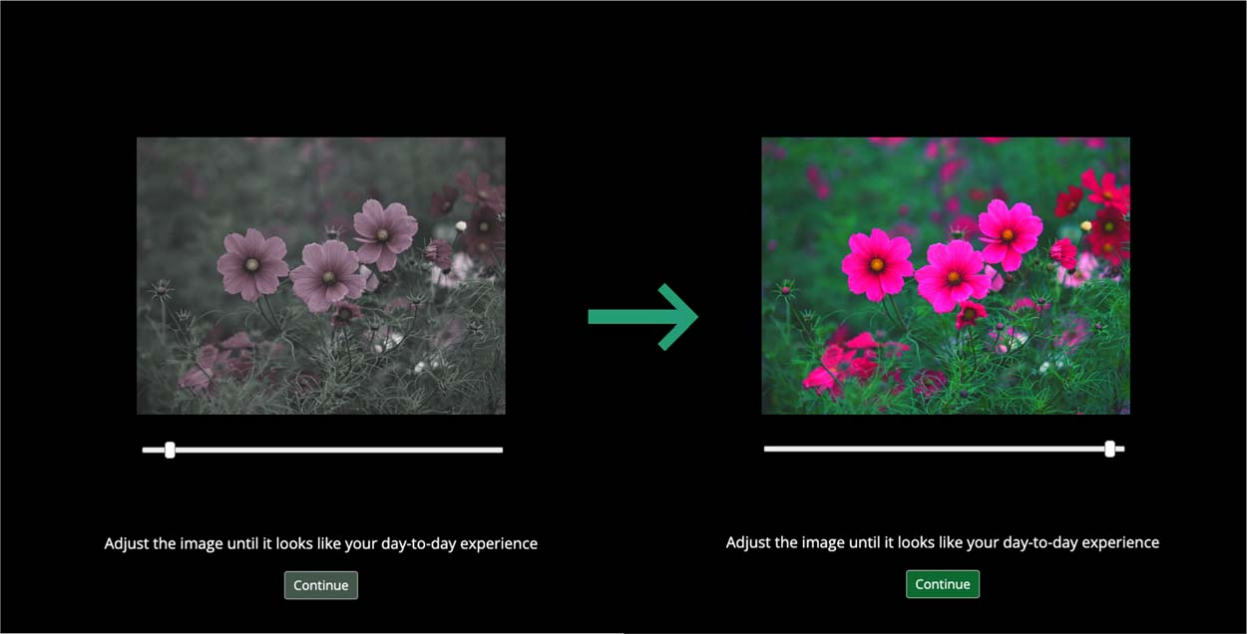
Illustration of the image adjustment task in the saturation condition. Participants adjust the slider below the image and thus set the saturation level of the image. This example shows the same image with extremely low (~20%) on the left side and high (~180%) saturation settings on the right side.

The images appear in the middle of the screen with a slider below them. We ask the participant to adjust the images using the slider until it looks like their day-to-day experience (Fig. 6). The images are manipulated based on the slider position converted into a percentage. This means when the slider is placed at the position corresponding to 100, then the image appears at 100% saturation/contrast level (i.e. the original image). Accordingly, when the slider is in the position corresponding to 25, then the image appears with 25% of the original saturation/contrast level. We have systematically varied the minimal and maximal positions of the slider. The widest possible range covered is between 10% and 190% of the original saturation/contrast level.

Once the participants are satisfied with the contrast or saturation level of the image, they are asked to click on the ‘Continue’ button at the bottom of the screen.

For each trial, we record the number of adjustments, response times, and the final slider positions. To minimize response biases, we pseudo-randomized the slider starting position between 50%, 100%, and 150% as well as the direction of increasing slider values. The stimuli are presented in a randomized order first and then a second time in the same order to assess the consistency across responses within participants (e.g. Fig. S3). The experiment is hosted on MindProbe.eu using JATOS (version 3.7.x) (Lange et al. 2015).

#### Cambridge Depersonalisation Scale

After the experimental task, we ask the participants to complete questionnaires on DP/DR experiences. We will use the Cambridge Depersonalisation Scale (CDS), which is a self-rated questionnaire measuring the frequency and duration of various depersonalization and derealization symptoms during the preceding 6 months (Sierra and Berrios 2000). Later studies used this scale to assess trait DP/DR, thus we are referring to the Cambridge Depersonalisation Scale as CDS-trait (Hunter et al. 2005). The scale includes 29 items. Each item lists seven options for the duration (0: Not Applicable, 1: Few seconds, 2: Few minutes, 3: Few hours, 4: About a day, 5: More than a day, 6: More than a week) and five options for frequency (0: Never, 1: Rarely, 2: Often, 3: Very Often, 4: All the Time) of the DP/DR episode. Based on the option selected, the scores are summed out of 10 for each item. The total CDS-trait scores range from 0 to 290. In addition, we have included one catch question with special instructions to check if the respondent was reading the items carefully.

The participants will also complete the state version of CDS. The CDS-state aims to assess the severity of DP/DR symptoms the participants are experiencing at the moment while filling out the questionnaire (Medford et al. 2003). It consists of 22 items, which are evaluated on a visual analogue scale based on the intensity of the experience. For our study, we opted to implement a circular response scale ranging from 0% to 100% in steps of 10. The total CDS-state scores range from 0 to 220. After the CDS scores, we also collect the demographic data and details of any psychiatric diagnosis. We will use Qualtrics (Provo, UT, https://www.qualtrics.com) to distribute the questionnaires to the participants.

It is possible that the CDS-state measure might be affected by the preceding task administration, and thus it might misrepresent the population distribution of the CDS-state. However, performing the CDS prior to the task is likely to influence the task outcomes. To better understand this order effect, we will take the following approach. First, we will run the experiments starting with the task and then completing CDS-state until we reach our decision criteria. If we find supporting evidence for H1 in any condition, then we complete a second round of experiment in that condition using the same sample size as in the first round. But this time recording CDS-state ‘before the task’. This would allow us to check if there is an increase in DP/DR symptoms during the completion of the task.

At the time of Stage 2 manuscript writing, we note that because H1 was not supported, we did not repeat the second round of the experiment in any condition.

### Procedure

We will recruit participants with Prolific (www.prolific.co). Eligibility criteria include being fluent in English and using desktop devices to do the experiment. Before beginning the experimental task, participants are given an explanatory statement and the consent form. The experiment only proceeded when the participants gave their informed consent by pressing a key. Then, we instruct them to (1) make their environment as dark as possible by switching off the lights and/or closing their curtains, (2) adjust the screen brightness to maximum, and (3) turn off any colour adjustment on the screen, such as NightMode, NightShift, or F.lux. After that, the participants will complete one practice trial and then proceed to the experiment. For Experiment 1 (the realness-rating task), the participant will receive compensation of £5.63 for their time (45 min). For Experiment 2 (the image adjustment task), the compensation will be £4.50 for 30 min. The project was approved by the Monash University Human Resource Ethics Committee under ID 17674.

### Statistical analysis

For all data processing and statistical analysis, we use R version 4.2.0 or higher (R Project for Statistical Computing, RRID:SCR_001905).

#### Analysis plan

We will analyse the data using Bayesian mixed-effects modelling with the brms package (Bürkner 2017). We will set up two models for each experiment, a full model, which includes our derealization measure, and a reduced model, which does not. We will compare the reduced and full models using Bayes Factors (BF) to assess which model is better supported by the data. In line with the recommendation of Schad et al. (2022), first, we describe our models below. Then, we will describe the priors which we have selected using our pilot data and prior predictive checks. We opt to do sequential data collection (see details in the sample size estimation section) with an upper limit of 500 participants per experiment and the decision criteria of BF10 or BF1/10. Once we reach either our upper limit of participants or the decision criteria are met, we will stop the data collection.

#### Model description

In the case of the realness-rating task (Experiment 1), the outcome variable is the realness ratings transformed to be in the range between 1 and 8, and we will model it as an ordered categorical variable using the cumulative link function. The random effects include random intercepts for stimuli and participants as well as random slopes for the manipulation level for both stimuli and participants. When it comes to the fixed effects, we will model the manipulation levels with a polynomial term. This is because the pilot data show that the lowest and highest levels of image manipulation tend to result in lower realness ratings compared to the original image. We have used the pilot data to implement a forward selection approach in order to identify what degree of polynomial term provides the largest improvement on the model (see details in Fig. S2 in the supplementary materials). Based on this, we will use a quadratic polynomial term for the manipulation level in both saturation and contrast models, and we will also include the quadratic term in the random effect structure. These model parameters will be used in the reduced model. For the full model, we will extend the reduced models with the inclusion of CDS-state scores as the fixed effect and adding an interaction term with the manipulation level.

*Full model:*


\begin{align*} Realness\ Rating\sim poly\left( Level,2\right)\ast\, & CDSstate+\left(1+ poly\left( Level,2\right)\mid ppt\right)\\& +\left(1+ poly\left( Level,2\right)\mid stim\right) \end{align*}

In the case of the image adjustment experiment (Experiment 2), the outcome variable is the response of the participant, which is obtained from the final slider position, and thus, we treat it as a continuous variable. Here, the reduced model includes the random intercepts (stimuli and participants) only, and the full model includes CDS-state scores. In addition, we plan to complete exploratory analyses by including additional fixed effects, specifically CDS-trait scores and image content categories.

*Full model:*


$$ Vividness\ response\sim CDSstate+\left(1\mid participant\right)+\left(1\mid stimulus\right) $$

#### Priors

We opted to use regularizing priors, which down-weight unlikely values (Vasishth et al. 2022). We have also completed prior predictive checks on the outcome variables informed by the pilot data. For the realness-rating task, we will use the default student-t prior (3, 0, 2.5) for the intercept. For the fixed effect, we use Normal (0,1) prior. For all random effects variance components and the residual standard deviation, we will use exponential (1) priors. For the correlation matrices in the random effects, we use LKJ(2) priors. In the image adjustment paradigm models, we use Normal (100,30) for the intercept, Normal (0,1) prior for the fixed effects, and exponential (0.1) priors for random effects variance components and the residual standard deviation.

### Sample size estimation

For all four experiments, we will follow a sequential design with an upper bound of 500 participants based on financial limitations (Schönbrodt and Wagenmakers 2018). For the realness-rating task (Experiment 1), we will start with 100 participants due to the complexity of the random effect structure of our proposed models. We will increase the sample size by 100 participants sequentially until we reach the Bayes Factor of 10 in favour of either the null model or the full model, or we reach our upper bound of 500 participants. In the case of the image adjustment experiment (Experiment 2), we will start with 50 participants as the initial sample and then increase the sample size by 50 participants sequentially. We will stop data collection when we reach the Bayes Factor of 10 in favour of either the null model or the full model, or we reach our upper bound of 500 participants. We decided on the starting sample size and the step size of the increase based on practical considerations for online experiments, including time and cost efficiency. For the simulation of the proposed sample size, see Supplementary Material. See Table 1 for the summary of the study design. 

## Results

We have collected the data in April and May 2024 after receiving in principle acceptance in February 2024, with the full sample described in Table 2. As described in the Sample size estimation, we collected data in a sequential design. For Experiment 1 (realness rating), we first collected 100 participants after attention checks for both saturation and contrast conditions. In each case, we reached BF_01_ >10 with 100 participants. In Experiment 2 (image adjustment), we collected data from 50 participants and reached BF_01_ >10 after 50 and 75 participants in the contrast and the saturation condition.

**Table 1:** Study design template

Question	Hypothesis	Sampling plan	Analysis plan	Rationale for deciding the sensitivity of the test for confirming or disconfirming the hypothesis	Interpretation given different outcomes	Theory that could be shown wrong by the outcomes
**Experiment 1** Does the relationship between **realness judgements** and **vividness** change when people experience DP/DR? Image manipulation conditions: —saturation —contrast	**(H1)** Current DP/DR experiences influence realness ratings	Sequential sampling (with an upper limit of 500 ppts.) until decision criterion is reached (Bayes Factor of 10 supporting H1 or 1/10 supporting H0)	Mixed model comparison *via* Bayes Factor estimation (based on bridge sampling) The full models (i.e. H1) include CDS-state while the null models (i.e. H0) do not	We chose the Bayes Factor of 10 as our decision criterion. In the data collection simulations, we used a standardized beta coefficient of 0.3 for the fixed effect of CDS-state (without interaction term) in Experiment 1 and 0.1 for the fixed effect of CDS-state in Experiment 2 as the smallest effect size of interest. We chose these effect sizes based on our subjective judgement on what we consider as an interesting effect in these experiments	**H1:** Experiencing derealization changes the association between apparent vividness and realness **H0:** Experiencing derealization does not affect the association between apparent vividness and realness	Unreal visual experiences in derealization are independent of visual cues such as changes in apparent vividness
**Experiment 2** Do people experiencing DP/DR differ in the **vividness** of their day-to-day (*‘real’*) experience? Image manipulation conditions:—saturation—contrast	**(H1)** Current DP/DR experiences influence what level of vividness people select to reproduce their day-to-day visual experience.				**H1:** Experiencing derealization changes what level of vividness people consider as matching to their usual experiences **H0:** Experiencing derealization does not affect what level of vividness people consider as matching to their usual experiences	

**Table 2:** Participant demographics

Experiment	Condition	Recruited ppts.	Retained ppts. (average age ± SD)	Gender distribution (female/male/non-binary/preferred not to respond)	Outcome
Experiment 1	Saturation	106	100 (29.31 ± 8.4)	47f/49m/3nb/1nr	BF_01_ > 1000
	Contrast	110	100 (30.39 ± 9.18)	47f/50m/2nb/1nr	BF_01_ > 100
Experiment 2	Saturation	93	75 (32.44 ± 9.62)	26f/49m/0nb/0nr	BF_01_ > 15
	Contrast	55	50 (29.04 ± 8.22)	10f/39m/1nb/0nr	BF_01_ > 14

**Figure 7:**
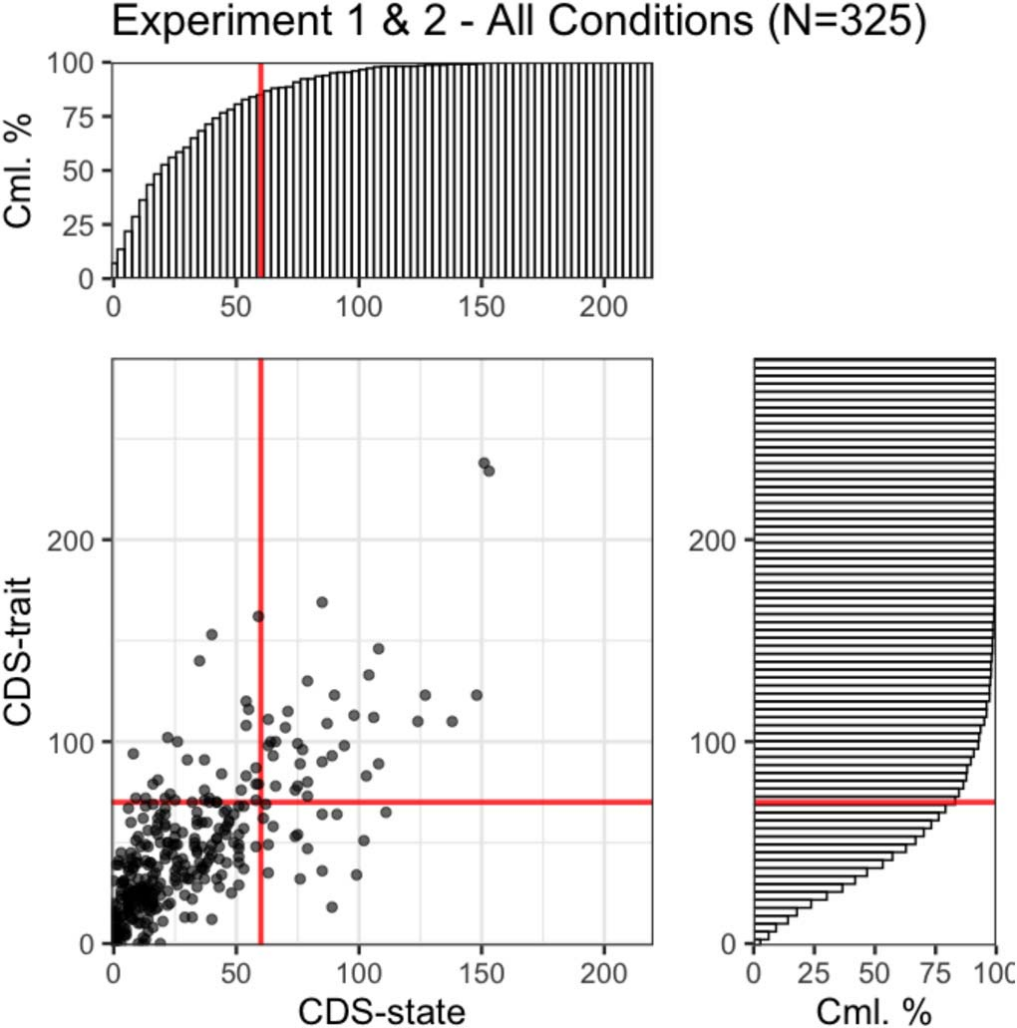
CDS-state (mean: 31.26 ± 30.16 SD) and CDS-trait score (mean: 46.71 ± 35.48 SD) distributions across all experiments. The lines indicate 70 for CDS-trait, which was proposed as clinically relevant cut-off score (Sierra and Berrios 2000), and 60 for CDS-state based on pretreatment depersonalization disorder patient scores (mean: 38.8 ± 21.8 SD) reported in Hunter et al. (2005). In the full sample, 50 participants obtained CDS-state scores ≥60 (i.e. 15% of the participants) and 66 participants obtained CDS-trait scores ≥70 (i.e. 20% of the participants). CDS-state and CDS-trait scores are strongly correlated (mode of posterior distribution of β coefficient = 0.74, 95% HDI [0.66, 0.81]).

The CDS score distribution for each experimental condition is presented in Fig. 7.

### Registered analysis

Using the priors and model parameters described above, we have fitted separate models with and without including the CDS-state scores and compared them *via* Bayes Factors estimated through bridge sampling. We have used 4 MCMC chains with 10 000 iterations (2000 warmup) to fit each model to achieve stable Bayes Factor estimates (Schad et al. 2022). The chain convergence and resolution were satisfactory in all of the models, and we have made the fitted models available in our Open Science Framework (OSF) repository. We have calculated the Bayes Factor by comparing the same two models 10 times to check the consistency of the estimate, and so we report the mean Bayes Factor estimate with standard deviations. We also report the posterior probabilities of the models calculated with uniform prior model probabilities. Finally, we describe the posterior distribution of the coefficients using 95% high density intervals (HDI) and the posterior mode as point estimates obtained with the tidybayes package (Kay and Mastny 2024).

**Figure 8:**
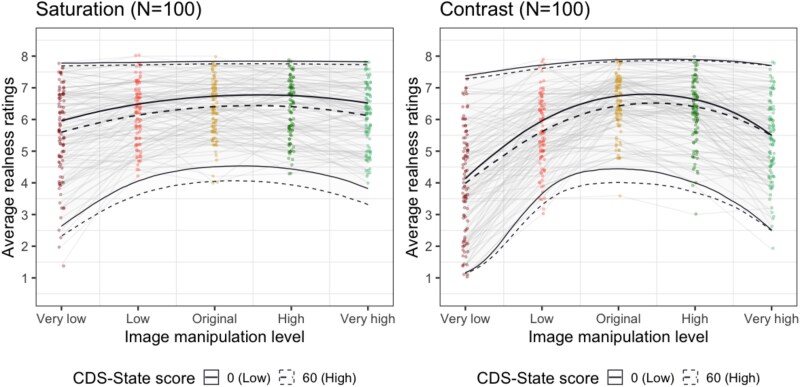
CDS-state did not affect realness ratings (Experiment 1). The individual dots and faint grey lines indicate average realness ratings per participant in each image manipulation level. The thick solid and dotted lines indicate the posterior estimates of the fitted models with 95% HDI (high density intervals), predicted to have 0 or 60 CDS-state score. The HDI overlaps substantially, showing that CDS-state scores are minimally relevant for the average realness ratings.

In all of the four registered tests, the BF supported the null model, which did not include the CDS-state scores. Specifically, in Experiment 1, we obtained extremely strong evidence for H0 according to the classification scheme by Lee and Wagenmakers (2014). This is most apparent by almost complete overlap of HDI across all levels of image manipulation for the predicted participants with CDS-state score of 0 or 60 (Fig. 8). BF were as follows: saturation condition: BF_01_ = 1358.81 ± 174.47 SD, with posterior probabilities >0.99 for null model; contrast condition: BF_01_ = 131.28 ± 18.78 SD, with posterior probabilities of >0.99 for null model. The CDS-state scores showed an overall small decrease in realness ratings as indicated by the fixed effect coefficient (saturation: β_CDS-State_ = −0.22, 95% HDI [−0.46, 0.01]; contrast: β_CDS-State_ = −0.20, 95% HDI [−0.47, 0.04]) and little-to-no indication of any interaction with the image manipulation level for both saturation and contrast conditions (all β estimates for interactions are between −0.02 and 0.09; see Table 3).

**Table 3:** Posterior mode estimates and 95% Highest Density Intervals (HDIs) for fixed effects from the Bayesian mixed-effects models.

Experiment 1—Saturation	
Variable	Posterior mode and 95% HDI
poly(Image manipulation Level,1)	0.22 [0.13, 0.30]
poly(Image manipulation Level,2)	−0.32 [−0.39, −0.24]
CDS-State	−0.22 [−0.46, 0.01]
CDS-State:poly(Image manipulation Level,1)	−0.02 [−0.09, 0.06]
CDS-State:poly(Image manipulation Level,2)	0.00 [−0.07, 0.07]
Experiment 1—Contrast	
Variable	Posterior mode and 95% HDI
poly(Image manipulation Level,1)	0.55 [0.41, 0.72]
poly(Image manipulation Level,2)	−1.05 [−1.19, −0.90]
CDS-State	−0.20 [−0.47, 0.04]
CDS-State:poly(Image manipulation Level,1)	0.02 [−0.11, 0.16]
CDS-State:poly(Image manipulation Level,2)	0.09 [−0.05, 0.22]
Experiment 2—Saturation	
Variable	Posterior mode and 95% HDI
Intercept	117.11 [110.50, 123.2]
CDS-State	−0.02 [−0.14, 0.10]
Experiment 2—Contrast	
Variable	Posterior mode and 95% HDI
Intercept	111.12 [105.64, 116.44]
CDS-State	−0.03 [−0.15, 0.09]

**Figure 9:**
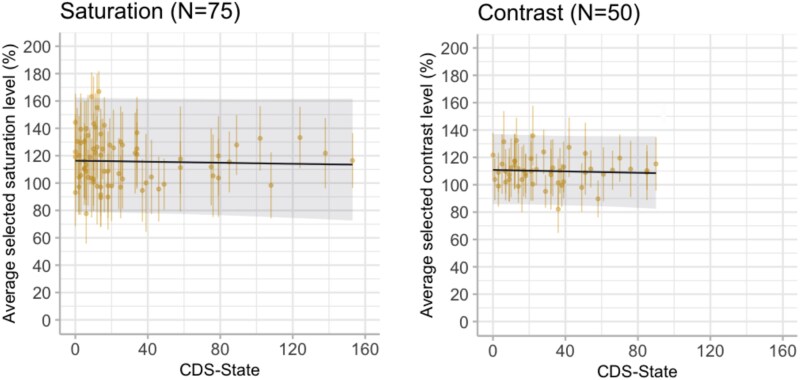
CDS-state showed no effect on the average vividness levels selected by the participants in the image adjustment task (Experiment 2). The *y*-axes are the level of saturation or contrast that participants selected to represent their day-to-day experiences. Each dot shows the average response of a participant with error bars indicating the SDs across 40 different stimuli responses. The dark line indicates the posterior estimates of the fitted models with 95% HDI as the shaded area.

In Experiment 2, we tested if participants’ active interaction with the image would reveal an altered relationship between perceived vividness and DP/DR symptom severity. Once again, we obtained strong evidence supporting the null models suggesting that CDS-state scores did not influence image adjustments. Bayes Factors preferring the null models are as follows: saturation condition: BF_01_ = 15.75 ± 0.44 SD, with posterior probability of 1 for null model; contrast condition: BF_01_ = 14.55 ± 0.20 SD, with average posterior probability of 0.94 for null model. As can be seen in Fig. 9 and Table 3, the fixed effect coefficients of CDS-state show central tendencies near zero (β_CDS-State_ = −0.02, 95% HDI [−0.14, 0.10] in the saturation condition and β_CDS-State_ = −0.03, 95% HDI [−0.15, 0.09] in the contrast condition).

Regardless of CDS scores, participants on average selected ~10%–20% higher saturation and contrast levels than the original image. We quantified this as the intercept of the model with the posterior intercept coefficient estimates being: saturation condition: 117.11, 95% HDI [110.5, 123.2]; contrast condition: 111.12, 95% HDI [105.64, 116.44] with 100 indicating 100% of saturation/contrast, which is the original image. In other words, participants tended to produce more saturated and higher contrast images to represent their everyday experience.

### Exploratory analysis

The registered analyses showed that current DP/DR experiences indicated by the CDS-state scores only exert a small influence on the realness ratings in Experiment 1 and have no effect on image adjustments in Experiment 2. These findings were consistent across two versions of vividness manipulations, contrast and saturation.

To explore potential factors that could explain these null results, we further examined three questions. First, does the effect of CDS-state remain negligible even when we only include CDS-state items describing perceptual alterations? Second, are there any image categories that are more sensitive to detect the effect of CDS-state than other categories? Third, can persistent DP/DR symptoms, measured *via* the trait version of CDS, be a better indication of altered vividness experiences than the state version of CDS?

#### Is a subset of the CDS-state scores more correlated with altered vividness than the rest of the scores?

DP/DR symptoms are diverse and items describing changes in the visual perceptual experience make up only a small portion of the entire scale of the full CDS-state score. To test if a subset of the CDS-state scores more correlated with altered vividness than the rest of the scores, we selected all items specifically describing visual changes. That is, we selected three items: item 2, ‘Things around me are now looking “flat” or “lifeless”, as if I were looking at a picture’; item 10, ‘My surroundings are feeling detached or unreal, as if there was a veil or a fog between me and the outside world’; and item 14, ‘Objects around me are looking smaller or further away.’ We fitted the same CDS-state models using only the sum of these item scores this time.

In Experiment 1, including only the CDS-state perceptual alterations scores resulted in a shrinkage in effect sizes in both conditions for fixed effects (saturation: β_CDS-StatePA_ = −0.19, 95% HDI [−0.42, 0.06]; contrast: β_CDS-StatePA_ = −0.08, 95% HDI [−0.35, 0.18]) as well as interactions (all β estimates for interactions are between −0.04 and 0.06). Therefore, we did not compare these models *via* Bayes Factors.

**Figure 10:**
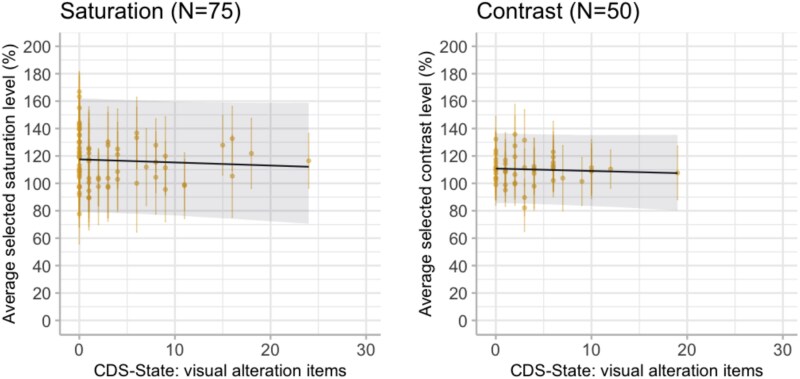
The effect of derealization symptoms measured *via* the perceptual alteration items (3 items with a minimum value of 0 and maximum value of 10) of CDS-state remained negligible on the selected vividness in the image adjustment task (Experiment 2). Each dot shows the average response of a participant with error bars indicating the SDs across 40 different stimuli responses. The dark line shows the posterior estimates of the fitted models with 95% HDI as the shaded area.

In Experiment 2, we found that these three items scores lead to an increase in the effect sizes (saturation: β_CDS-StatePA_ = −0.15, 95% HDI [−0.96, 0.51]; contrast: β_CDS-StatePA_ *=* −0.13, 95% HDI [−0.88, 0.52]). So, we compared these models with the null models. We found anecdotal evidence for the null models in this case with the following Bayes Factors. Saturation condition: BF_01_ = 2.21 ± 0.03 SD, with the posterior probabilities favouring the null model (0.69 for null model). Contrast condition: BF_01_ = 2.55 ± 0.05 SD, with the posterior probabilities favouring the null model (0.72 for null model). We show the average selected vividness levels in relation to the total scores of the perceptual alterations items in Fig. 10.

#### Is there any image category that was more sensitive to detect the effect of CDS-state than other categories?

Since Yokokawa et al. (2018) have used only the flower image content category in their study which included realness judgements based on apparent vividness, we explored if this category invokes distinct response strategies in our tasks.

We tested the impact of including image content categories (i.e. flowers, indoor scene without people, outdoor scene without people, scenes with people) into our models in both Experiments 1 and 2. In Experiment 1, the image content categories did show a differential effect on the ratings as indicated by the BFs showing extremely strong evidence in favour of including the image category into the models with BF_10_ >1000 in the saturation condition and BF_10_ = 633 ± 112 in the contrast condition. Specifically, the images depicting flowers were consistently rated as less real compared to all other image categories, and this effect was larger in the saturation manipulation condition. Compared to flower image category, the coefficients were β_Indoor_ = 0.62, 95% HDI [0.31, 0.89], β_Outdoor_ = 0.52, 95% HDI [0.22, 0.78], β_People_ = 1.07, 95% HDI [0.81, 1.38] in saturation condition; and β_Indoor_ = 0.13, 95% HDI [−0.14, 0.38], β_Outdoor_ = 0.05, 95% HDI [−0.23, 0.30], β_People_ = 0.62, 95% HDI [0.36, 0.90] in the contrast condition. So, realness ratings may vary based on the type of image content that is presented.

Importantly, however, these category effects did not strongly interact with the CDS-state scores. β Estimates for interactions were in the range of −0.12 to −0.05 for saturation and of −0.03 and −0.02 for contrast. For the sake of brevity, we listed details of the model coefficients and HDIs in the supplementary materials (S7).

In the case of Experiment 2, the inclusion of the image content categories resulted in moderate evidence for full model with variable BF estimates in the saturation condition (BF_H1_ = 6.26 ± 19.06 SD) with the posterior probabilities slightly favouring the null model (0.53 for null model) and strong evidence for the full model in the contrast condition (BF_H1_ = 26.23 ± 53.34 SD, with posterior probability of 0.64 for full model). Compared to flower images, the coefficients were β_Indoor_ = −0.18, 95% HDI [−2.00, 1.83], β_Outdoor_ = −0.14, 95% HDI [−1.94, 1.87], β_People_ = 0.36, 95% HDI [−1.55, 2.30] in saturation condition; and β_Indoor_ = −0.14, 95% HDI [−2.09, 1.68], β_Outdoor_ = 0.05, 95% HDI [−1.86, 1.93], β_People_ = 0.16, 95% HDI [−1.71, 2.01] in the contrast condition.

Once again, none of these image category effects interacted strongly with CDS-state. Coefficients for the interactions showed central tendencies near zero with 95% HDIs within the range of −0.12 to 0.8 in both conditions.

These results suggest that the effect of image content is relatively weak with high uncertainty based on our data when it comes to what levels of vividness people select when reproducing everyday experiences. Thus, image content seems more consequential for realness ratings compared to active vividness manipulation. Still, CDS-state scores showed very limited interaction with image content categories across all experiments.

#### Is CDS-trait more strongly associated with the vividness alteration than CDS-state?

Since in Experiment 1 (see Fig. 8) we observed a small negative shift in the ratings across all image manipulation levels as the CDS-state score increased, we tested if this shift could be the sign of an altered response tendency in people with frequent DP/DR experiences over a prolonged period. So, we repeated our analysis with the trait version of the CDS instead of the state version with everything else remaining the same.

**Figure 11:**
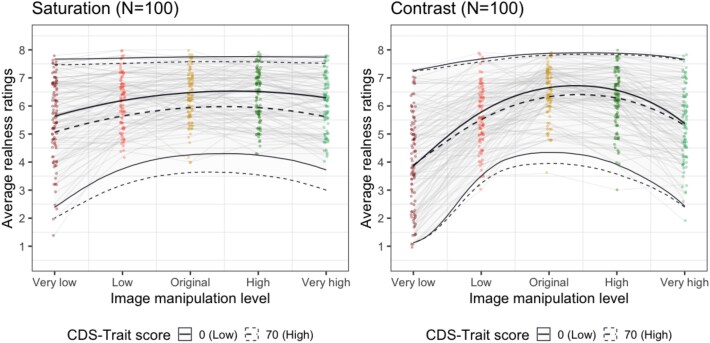
CDS-trait effect on realness ratings. The dots indicate average realness ratings per participant in each image manipulation level in Experiment 1. The lines indicate the posterior estimates of the fitted models with 95% HDI.

In Experiment 1, we found a weak negative effect of CDS as a main effect (see Fig. 11) that was the largest in case of the saturation condition (saturation: β_CDS-Trait_ = −0.33, 95% HDI [−0.57, −0.10]; contrast: β_CDS-Trait_ = −0.21, 95% HDI [−0.46, 0.07]). Yet, the BFs still favoured the null models which did not include CDS-trait scores with BF_H0_ >1000 in the saturation condition and BF_H0_ >1000 in the contrast condition. While there may be an overall small negative shift in realness ratings in people experiencing derealization regardless of the presence of DP/DR symptoms, this effect is too small to improve the model fit evaluated *via* Bayes Factors. Since image content categories improved our models in Experiment 1, we included them as fixed effects in this analysis as well and found the same pattern of limited interaction between image categories and CDS-trait with β estimates between 0.16 and 0.03 for saturation and −0.09 and −0.02 for contrast.

In Experiment 2, CDS-trait coefficients showed central tendencies near zero in both conditions (saturation: β_CDS-Trait_ = −0.02, 95% HDI [−0.14, 0.09]; contrast: β_CDS-Trait_ = 0.01, 95% HDI [−0.10, 0.10]) and the BFs also favoured the null models (BF_H0_ > 1000). Additionally, all β coefficients between CDS-trait and image content categories were also centred near zero. Thus, CDS-trait, which is supposed to measure persistent DP/DR experiences, was not interacting with the selected vividness levels in any of the image categories.

## Discussion

In this registered report, we have provided a novel empirical examination of the relationship between the subjective realness and vividness levels of naturalistic images. Notably, we have tested the effect of derealization symptoms, operationalized by CDS scores on this relationship. Derealization experiences are often described in subjective reports to involve specific visual alterations, such as changes in colour intensity or clarity of the perceptual experience. Thus, we have hypothesized that apparent vividness could affect how real an image is perceived depending on the severity of current derealization experiences (i.e. CDS-state scores). Overall, across two separate tasks (realness ratings in Experiment 1 and image adjustment in Experiment 2) with two vividness manipulations (i.e. contrast and saturation), we found no evidence to support that DP/DR experiences affect what vividness levels are associated with subjective realness in non-clinical population. These findings provide empirical grounding for theoretical frameworks describing derealization as a metacognitive process independent of visual changes.

### Realness rating task (Experiment 1)

We expected that the subjective evaluation of realness of diverse, naturalistic images, with their contrast or saturation manipulated, would reveal a difference in people reporting DP/DR symptoms compared to those without such experiences. Contrary to H1, our results showed strong evidence against CDS scores affecting realness ratings. The findings remained the same even when solely testing the perceptual alteration items of the CDS-state scale. This suggests that even when participants specifically report changes in the current visual perception, such as their environment appears, as if they were looking at a picture, realness judgements of image stimuli remain unaffected. Thus, the association between the vividness of a specific visual object and its realness seems to be intact even when participants with high CDS scores claim that the entire perceptual experience of the external world is deemed as unreal (i.e. experiencing derealization).

### Image adjustment task (Experiment 2)

Since we instructed participants in Experiment 1 to use their day-to-day experiences as a reference for evaluating how real the images look, we wanted to examine if derealization affects the vividness of what people consider as day-to-day experience *via* prompting participants to interactively manipulate images. Contrary to H2, we again found strong evidence against CDS-state scores affecting the image adjustment. When we examined the perceptual alteration items specifically, we found slightly increased effect sizes; however, the Bayes Factors still supported the null models. This finding suggests that participants invoked similar beliefs about vividness when instructed to refer to day-to-day experiences regardless of the presence of DP/DR symptoms.

### Reconciling our findings with previous studies

Our realness task was partially inspired by Yokokawa et al. (2018), whose primary objective was to find the neural correlates of perceptual fadedness associated with feelings of unreality in healthy individuals. In their paradigm employing visual adaptation, participants were first presented with a highly (or less) saturated image, followed by a medium-saturated image. Their results showed that medium-saturated images presented after highly saturated images were rated as having lower realness and saturation compared to those presented after less saturated images. Our results align with their findings in that less saturated images were rated as less real. However, DP/DR experiences did not affect this relationship. Thus, while Yokokawa et al. (2018) proposed that the processes involved in evaluating the realness of an image may also be involved in unreal experiences in derealization, our findings challenge this interpretation. Additionally, Yokokawa et al. used images depicting flowers only and they have only manipulated the saturation levels of these images. We extended the image manipulations to include contrast levels and the image content categories with three other categories. Interestingly, we observed that flower images were rated as less real compared to all other image categories, especially in the saturation condition. Yet, image content categories showed no interaction with CDS scores, so it is unlikely that image content affects realness judgements differently when derealization symptoms are present. Given our null findings using a more generalizable task, we suggest that the process of evaluating the realness of a perceptual object, such as an image on a screen, can be done independently from evaluating the realness of experiences in general. In other words, even when people report experiencing their environment as unreal, they can still make comparable realness judgements about images to people without unreal experiences.

### Potential interpretations: I. Metacognitive accounts

Among the theories we discussed in the introduction, the ones positing a metacognitive account of derealization appear to be the most compatible with our findings. These theories suggest that evaluating realness of the visual experience is a process done independently of the specific input (e.g. Dokic and Martin 2017). While we proposed an interpretation for how a positive finding would fit into the metacognitive account (see Fig. 2c), in light of the current results we suggest that derealization experiences indeed can be decoupled from specific visual stimuli. Additionally, subjective realness has been linked with various factors besides visual input. Deroy and Rappe (2022) proposed that realness is also influenced by multisensory congruence, feelings of control, and subjective certainty. Gładziejewski (2022) argued that the sense of realness is supported by an interoceptive background state (i.e. interoceptive appraisal). Finally, Suzuki et al. (2023) noted the importance of sensorimotor coupling and embodiment besides sensory factors in creating the sense of presence, which becomes disrupted in derealization. Altered metacognition may also affect the evaluation of the abovementioned factors such as interoception or sensorimotor integration. Thus, a metacognitive account can be aligned with most existing theoretical proposals. While our experiments did not assess factors beyond vividness, the results support that the altered realness experiences reported in DP/DR involve more than pure visual changes.

### Potential interpretations: II. Issues of measurement

We also need to consider if we could reliably measure DP/DR experiences. The CDS-state has been used to assess DP/DR symptoms and measure the efficacy of interventions across multiple studies in both clinical and non-clinical populations, so this measure is well established within the field (Hunter et al. 2005; Somer et al. 2013; Jay et al. 2014; Medford et al. 2016; Millman et al. 2022; Peckmann et al. 2022). In addition, a recent systematic review concluded that the CDS-trait exhibits high internal consistency, structural validity, and reliability (Wainipitapong et al. 2025). Thus, CDS scores appear to be an appropriate measure of DP/DR experiences.

While our study did not target individuals with severe DP/DR symptoms specifically, our sample included a substantial number of people with scores suggesting clinically relevant DP/DR symptom severity in both the state and the trait version of the CDS (Fig. 7). Similar mean and standard deviation scores have been reported in DP/DR patients in a few other studies (Schoenberg et al. 2012; Jay et al. 2014). In addition, a recent online study (Ciaunica et al. 2022c) which recruited 622 online participants through Prolific also reported similar average CDS-trait score of 49.3 (SD = 35.9), comparable to what we found in our sample. Furthermore, a recent meta-analysis investigated the prevalence of dissociative symptoms in student populations and found that 16.6% of students report pathological levels of dissociative experiences based on data from self-assessment (Kate et al. 2020). Thus, our online participant pool exhibited similar DP/DR severity as to what we would expect in the general population with clinically relevant symptom severity also represented in the sample. Still, our access to participants with high levels of DP/DR experiences was limited, so the data may not fully represent the variability and severity of derealization symptoms found in clinical populations, raising questions about the generalizability of our findings to patients. Yet, there is currently no evidence showing a difference between the derealization symptoms of patients and undiagnosed individuals with DP/DR experiences.

We also need to consider the potential limitations of our experimental paradigms. One potential weakness of our tasks was that they were completed online. Online experiments can only exert limited control over participants’ engagement and the environment in which they completed the task. However, recent registered studies using colour similarity in adults (Zeleznikow-Johnston et al. 2023) and children (Moriguchi et al. 2025) as well as colourful natural images (Qianchen et al. 2022) did not find any systematic qualitative difference in perceptual performances of participants tested online *versus* in-laboratory. Given these studies, we have little reason to question the sensitivity of our experimental tasks in online participants. Nonetheless, without explicit comparisons with online and in-laboratory participants, it remains possible that our null results may originate from the fact that our tasks were performed online, and it remains a potential issue to be examined in future studies. Given these, we predict that in-laboratory testing will result in the same results as ours. It will be especially interesting to confirm if the clinically confirmed DP/DR patients will produce the same results in the same range of behavioural effects as predicted from our regression models.

### Potential interpretations: III. The El Greco fallacy

As our study examined experiential change in the visual domain, the El Greco fallacy (Firestone and Scholl 2014) remains a critical issue to address when interpreting the findings. As we noted in the introduction (we also elaborated on the El Greco fallacy in the supplementary materials), one could argue that our null results support the notion that there is a consistent change in vividness perception during derealization because the perceptual change would affect all visual input equally including the image presented in the experiment leading to no difference in task performance. However, we maintain that this argument does not account for the key characteristic of DP/DR, which is people reporting—often spontaneously—that their experience is lacking in vividness. In both experiments, we asked the participants to use their day-to-day experience as the reference when completing the task. So, it is possible that people with persistent DP/DR symptoms use their perceptual experiences during derealization as their day-to-day experience. While we acknowledge this possibility, we argue that if this were the case, then we would expect to observe a difference between participants with high CDS-state and CDS-trait scores compared to participants with high CDS-state and low CDS-trait scores. The consistent null results make this interpretation very unlikely.

### Considerations for future research

Based on our findings, we may speculate that evaluating the realness of specific images or objects invokes different internal models or beliefs compared to internal models of the entire perceptual experience. Indeed, recent theories on perceptual reality monitoring proposed the existence of a hierarchical system evaluating information throughout the visual stream (Dijkstra et al. 2022; Sulfaro et al. 2023). Under these frameworks, we can conceptualize that the visual system decouples evaluating realness of an image from evaluating realness of the wider experience (also see Haun and Tononi 2025). Since our findings suggest that the evaluation of a distinct visual object such as an image remains intact in derealization, future studies may benefit from using virtual reality (VR) environments to modify the vividness of the entire visual experience. With this approach, we could decisively establish whether it is possible to capture the altered sense of realness using visual stimuli at all in derealization. Indeed, there is promising work using VR showing that visual features, such as graininess, can influence realness judgements (Drori et al. 2020). Furthermore, increased vividness through manipulating texture and lighting effects was found to enhance the sense of presence in VR environments in healthy participants (Huang et al. 2010). Thus, using VR could further elucidate if evaluating realness in derealization can be completely decoupled from the visual experience.

Our use of the term vividness was based on the recent conceptualization by Fazekas and colleagues (Fazekas et al. 2020; Fazekas 2024). As discussed in the introduction, they define vividness as a multifactorial quality of the experience involving subjective intensity features (i.e. contrast, saturation, and brightness), subjective specificity features (i.e. blurriness, graininess, and ambiguity), and temporal stability (Fazekas et al. 2020). (Note that ‘vividness’ of experience can be sometimes used to mean with relevance, intensity, emotional expressivity, richness, and memorability (Haustein et al. 2020).) Ambiguity of the critical terms, such as vividness, can lead to confusion among researchers, clinicians, and patients about how to characterize derealization symptoms based on such terms. Thus, future research would benefit from a more systematic approach when using the terms such as vividness. For example, instead of using vividness, specific features such as saturation can make phenomenological descriptions easier to translate to experimental predictions. We also note that when people describe their environment as ‘unreal’ or ‘dream-like’ together with reported changes in colourfulness or flatness, it is rarely clarified how drastic these changes are compared to ‘real’ or ‘not dream-like’ perceptual experiences. Future studies could obtain more detailed accounts on the extent of the perceptual changes in derealization by combining behavioural tasks such as ours with qualitative methods, e.g. descriptive experience sampling and micro-phenomenological interviews (Bass-Krueger et al. 2024). Such methodological improvement is necessary to facilitate more detailed, quantifiable, and systematic comparisons of phenomenal experience in an intersubjective manner. With that, we would be better able to shed light on potential diversities in phenomenology.

Finally, DP/DR symptoms are often triggered in response to stressful situations (Hoyer et al. 2013; Soffer-Dudek 2017). Since our study did not assess stress or anxiety levels and did not attempt to evoke any stress response, it would be informative to test the effect of a stress-inducing intervention on realness judgements. Potentially, this could reveal if metacognitive judgements such as realness assessment are more easily distorted under acute stress and explain the link between dissociative experiences and stress.

In conclusion, our study offers novel empirical data describing the relationship between subjective realness, vividness, and derealization symptoms. There are many references to vividness changes in derealization in qualitative studies (e.g. Pienkos and Sass 2022), and vividness is often mentioned in passing within theoretical works about derealization (e.g. Deroy and Rappe 2022; Gładziejewski 2022; Suzuki et al. 2023). Yet, our study could not detect any difference in task performance with saturation and contrast manipulations across varying levels of CDS scores. These results provide new directions for future empirical and theoretical works.

## Supplementary Material

Stage2_Manuscript_supplementary_niaf045

OPEN_SCIENCE_BADGE_APPLICATION_FORM_niaf045

## Data Availability

All data, materials, and codes underlying this article are available in the OSF repository, at https://doi.org/10.17605/OSF.IO/VEFSD.
