# Supplementary material for: Lifting the veil: probing altered visual perception in derealization
Source: Neurosci Conscious. 2025 Nov 23;2025(1):niaf045. doi: 10.1093/nc/niaf045 (PMC12640545; doi:10.1093/nc/niaf045)
Supplement: Stage2_Manuscript_supplementary_niaf045 [file stage2_manuscript_supplementary_niaf045.docx]

#
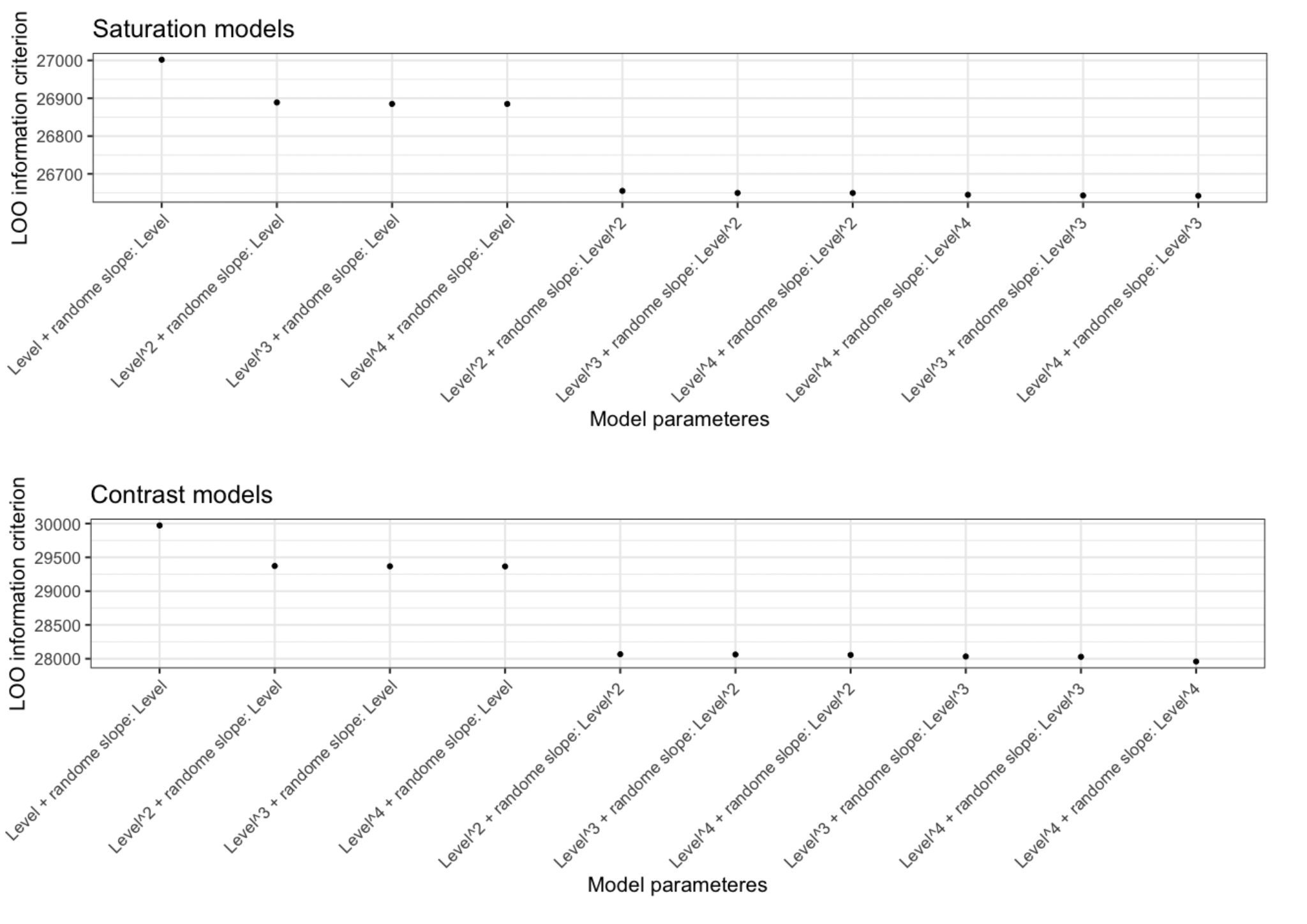
Supplementary Materials

*Figure S1. Model comparison using Leave-one-out cross-validation (LOO-CV) information criterion. We compared the reduced model with increasing degrees of the polynomial term on the fixed effect of the manipulation level variable as well as on the random slope of the manipulation level for both stimuli and participants.* Based on this analysis, we opted for using a quadratic polynomial term for the manipulation level in both saturation and contrast models, and we will also include the quadratic term in the random effect structure.

#

# S3. Considerations on the El Greco Fallacy

*Note this supplementary information is written at the time of Stage 1 acceptance and reflects our view at that time.*

Here we present an extended discussion of the El Greco Fallacy and argue for its limited relevance in the context of this research project.

Since our study aims to make inferences about difference between two populations (i.e., people experiencing derealisation versus people who do not) by manipulating a perceptual feature (i.e., vividness), the reasoning error known as the El Greco Fallacy (Firestone and Scholl, 2014) may raise some questions to address. In short, the core idea of the El Greco Fallacy is the following: If two groups are asked to report perceptual features of a target stimulus using a reporting tool displaying the same perceptual feature, then any difference found between these groups cannot be due to perceptual mechanisms. This is because the impact of a perceptual distortion must be cancelled out by the reporting tool, which is also affected by the same perceptual distortion. The fallacy occurs when a difference under these circumstances is attributed to perceptual mechanisms given the assumptions a) the perceptual mechanisms between the two groups are the same and b) the perceptual distortion is a constant-error distortion.

For example, if we were to ask people to report how vivid their current environment is via selecting the corresponding image from a set of images with different levels of vividness, then a perceptual shift in vividness would affect both the current environment as well as the images. Therefore, we could not detect the perceptual distortion even if it was present given assumptions a) and b) hold true. At the same time, if we saw a difference between two populations in selected vividness levels, it would be fallacious to conclude that there was a perceptual difference. Instead, the difference would be explained by non-perceptual mechanisms e.g., metacognitive differences, response bias and/or demand characteristics.

As described above, the direct application of the El Greco Fallacy strictly has limited relevance to our experiments because we do not ask participants to reproduce the vividness of their current environment.

In Experiment 1 we ask each participant to rate 100 different images, which we have manipulated to reach unnaturally high or low levels of vividness based on what they believe about their day-to-day experience and assess how real the stimuli look compared to this internal model of their day-to-day experience. So, we intend to invoke an evaluation of matchings between manipulated images and a template of what the world usually looks like.

In Experiment 2, we ask participants themselves to manipulate the images with the possibility for selecting unnaturally high or low levels of vividness. We use the following instruction: “Adjust the image until it looks like your day-to-day experience”. Here again we intend to invoke a comparison between the image and the template of what the world usually looks like but this comparison is done over and over again until the image is as close to matching the template as possible.

In both of our experiments, we use a selection of naturalistic images that are novel to the participants and that were produced under diverse lighting conditions, with different perspectives and composition.

Thus, our study does not aim to test vividness perception per se but the altered relationship between vividness and realness in derealisation. In other words, we consider vividness perception to be the characterization of F in Z = F(X), where X is the image features (such as saturation or contrast) and Z is vividness experience. We aim to describe G in Y = G(Z), where Y is realness experience and Z is vividness experience. Crucially, we do not claim to characterise Z so we will not make inferences about the vividness experience in derealisation based on our experiments. Instead, we presume that realness experience (i.e., Y) is different in derealisation since that is how individuals describe this state. Then, we hypothesise that the relationship between realness and vividness (i.e., G) differs between people with and without derealisation experiences. To test this hypothesis, we seek to manipulate vividness experience (Z) using our stimuli with varying image features.

#

# S5. Pilot data description


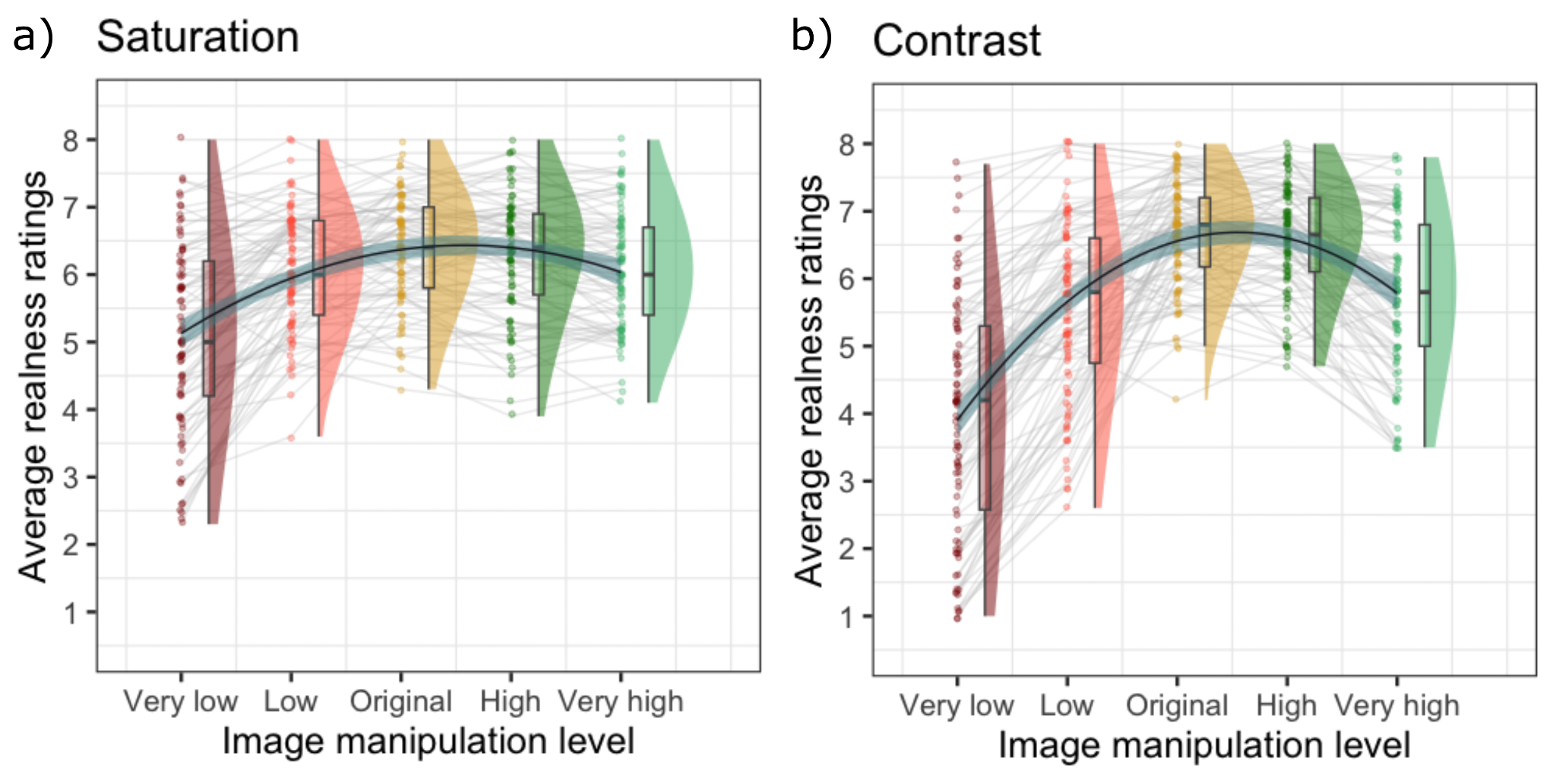
To demonstrate the feasibility of this research project, we have completed separate pilot experiments that included both the saturation and contrast conditions for the realness-rating task and the image adjustment task. The pilot experiments for the realness-rating task only included the CDS questionnaire for the trait, while the pilots of the image adjustment task included CDS questionnaire for both state and trait. For the realness-rating task, we recruited 100 participants for each pilot experiment and excluded any participants who failed to answer correctly to the catch question in the CDS-trait scale (4 exclusions in the saturation condition and 7 exclusions in the contrast condition). In addition, we excluded participants who responded incorrectly to more than 1 out of 10 catch trials in the task (i.e. 90% catch trial accuracy; additional 15 exclusions in the saturation condition and 5 exclusions in the contrast condition). Thus, the final participant number in the realness-rating pilots was N=81 in the saturation condition (Figure S2a) and N=88 in the contrast condition (Figure S2b). We plan to use the same exclusion criteria in the stage 2 analyses. We will report the results of the analyses using data with 100% catch-trial accuracy in the supplementary materials as a check of robustness.

*Figure S2. Pilot data from the realness-rating task. The dots represent the average realness rating per participant in each image manipulation level.*

In the pilot experiments of Experiment 2 the image adjustment task, we recruited 20 participants for each experiment. Here we excluded any participants who failed the catch question in the CDS-trait scale or did not fully finish the inventories. After this, the participant number was N=17 for the saturation condition and N=19 for the contrast condition (Figure S4). We have also assessed the correlation between the responses to the first and second presentation of the same image (i.e. double pass correlation). We calculated the participant’s double pass correlation coefficient using Pearson correlation and applied Fisher transformation on them. We opted to exclude any participant, whose transformed coefficient was 2 standard deviations below the median of the sample (see Figure S3). These criteria led to two additional exclusions in the pilot sample of contrast condition and no further exclusions in the saturation sample.


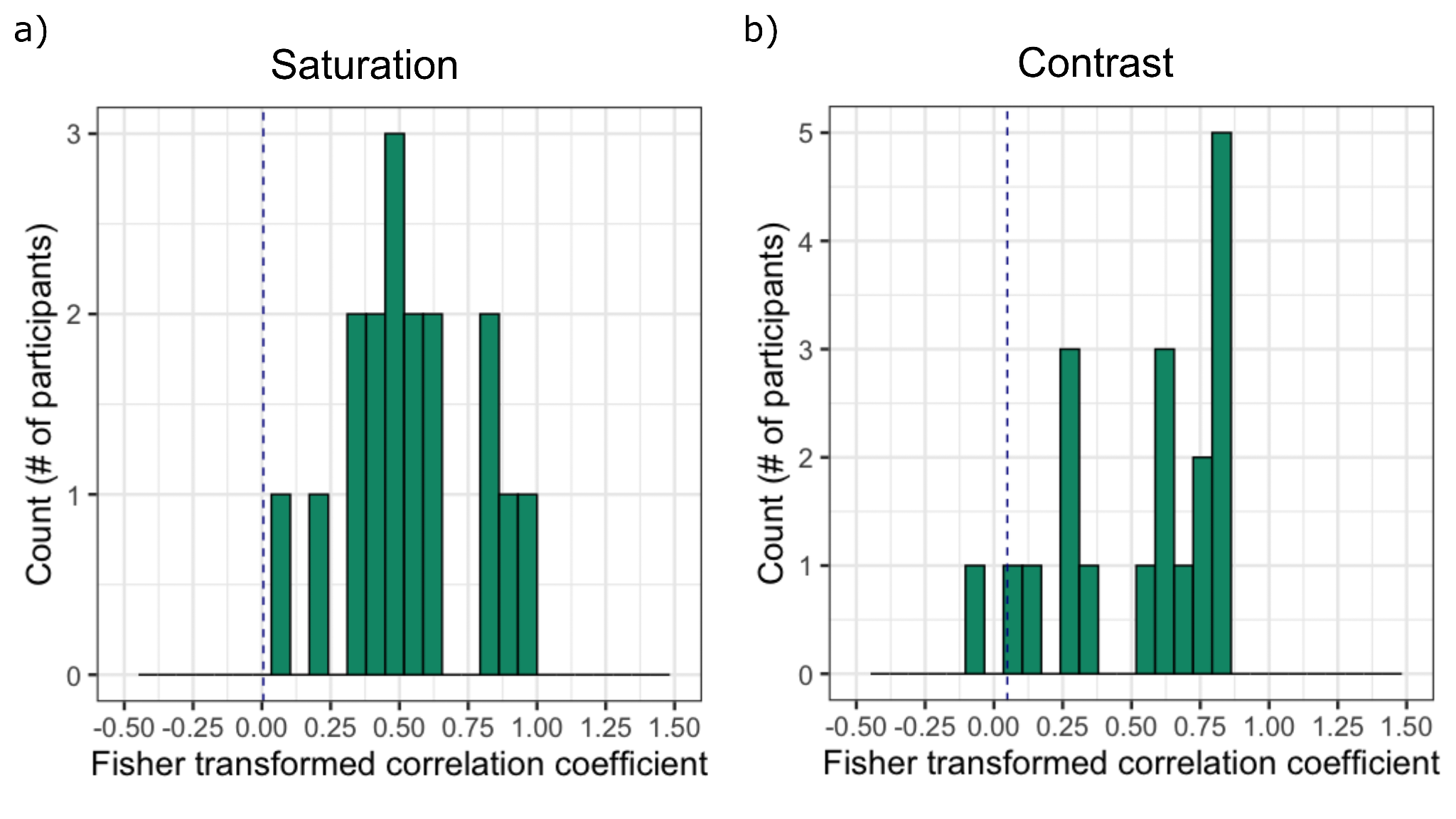


Figure S3. Fisher transformed double-pass Pearson correlation coefficients between the first and second presentation of the stimuli in the image adjustment task in the saturation (a) and contrast (b) pilot experiments before exclusion. The blue dashed line indicates 2 standard deviations below the median Fisher transformed coefficient in the pilot samples. These criteria resulted in two exclusions in the contrast conditions and no exclusions in the saturation condition.


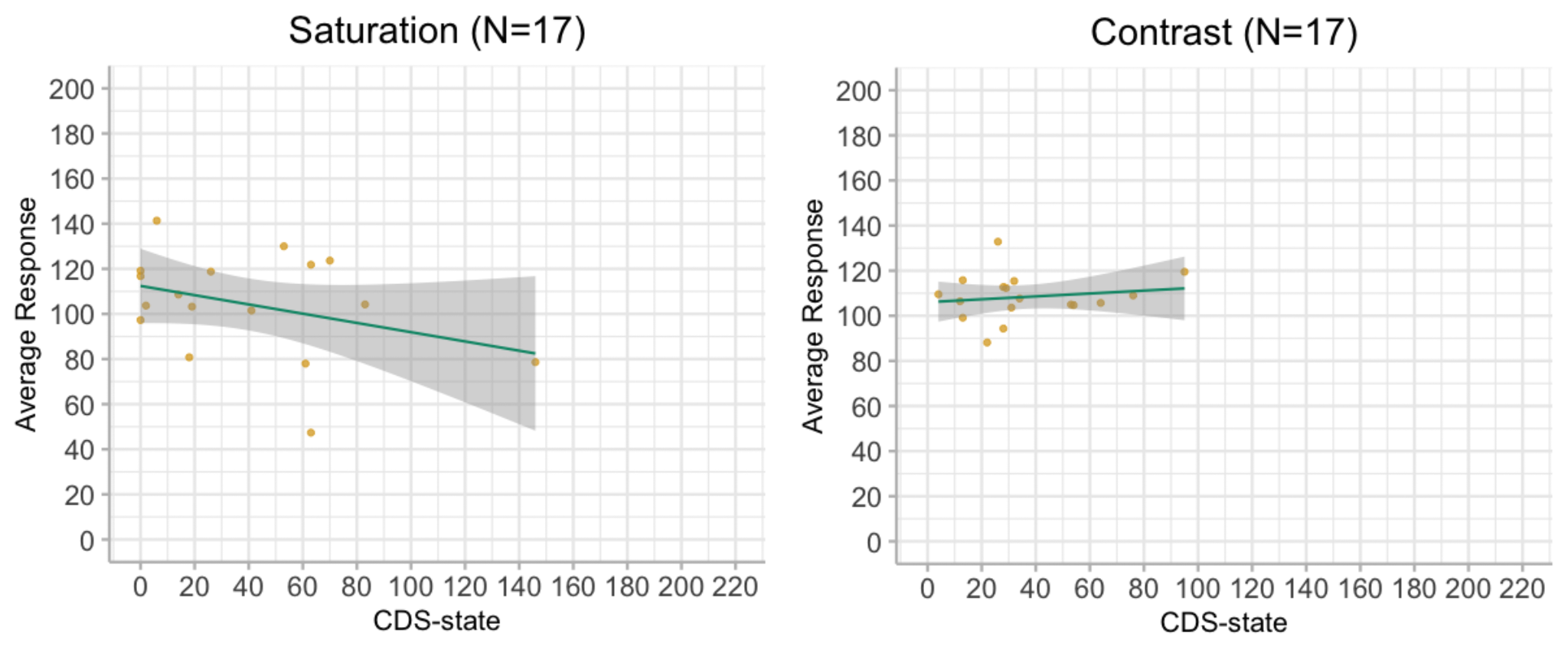
*Figure S4. Pilot data from the image adjustment task. The dots represent the average response per participant.*

#

# S6. Sample size simulation

To test if the range of the proposed sample size would be appropriate to measure the impact of CDS, we have simulated the sequential data collection process. In the case of the realness rating task, we have used slope coefficients of image manipulation level and random effect structure of the pilot data from the saturation condition in the simulations. For the image adjustment task, we have sampled the previously described priors to simulate the random effect structure (following the principles described by (Vasishth et al., 2022). For the CDS-state scores, we have used the mean and standard variation parameters of an unpublished dataset, which we have collected for a different research project, including 93 participants.

In the realness rating task, we chose 0.3 as the smallest regression coefficient of interest with the upper limit of 300 participants in order to reduce the required computation time. In the case of the image adjustment task, we chose 0.1 as the smallest regression coefficient of interest. We have also limited the upper limit of the participant number to 250.

We simulated 100 artificial datasets with the maximum number of participants and simulated the outcome variable using the regression coefficient of the CDS-state, which was set at the value of 0.3 for the realness rating task and 0.1 for the image adjustment task. Then we fitted the reduced and full model with the above-described priors on each dataset sequentially starting with a subset of the simulated participants’ data, and compared the models using Bayes Factor. If the decision criteria of BF_10_ or BF_01_ > 10 were met, then we moved to the next dataset. If the criteria were not met with the first subset of participants, then we added the next subset participants’ data according to the predetermined step-size and completed the model fitting and comparison again until reaching the upper limit of the simulations. Thus, we have effectively simulated 100 rounds of data collection. We have also completed the same steps for simulating null effects via setting the coefficient of CDS-state to zero. The results of the simulated data collections for the realness rating task are shown on Figure S5 and for the image adjustment paradigm in Figure S6.


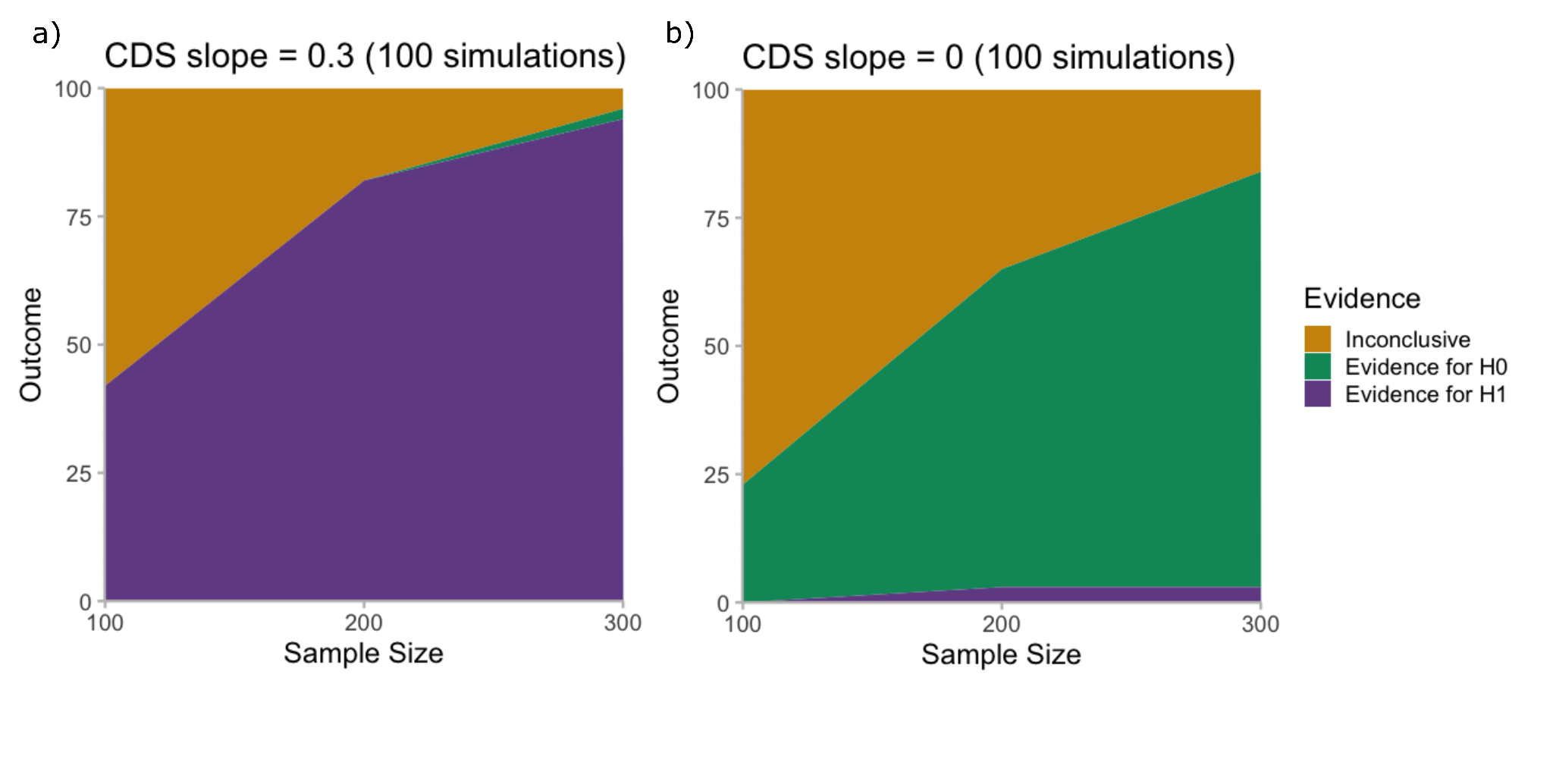
*Figure S5. The outcome of 100 simulated runs of data collection for Experiment 1 (realness rating task) using the decision criteria of*  BF_10_ or BF_01_ > 10  *and stopping at 300 participants. The full model in the simulations did not include an interaction term.*


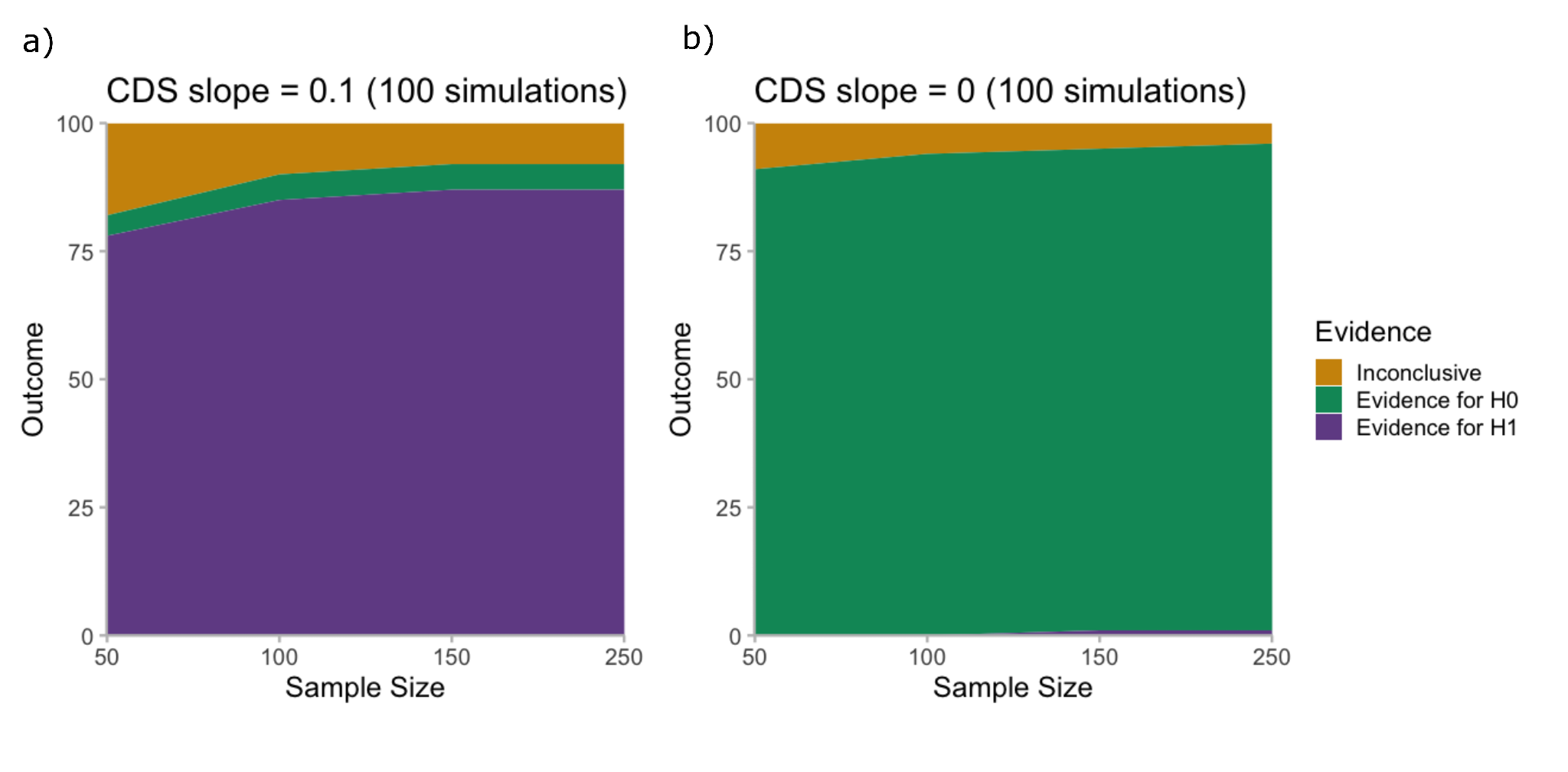


*Figure S6. The outcome of 100 simulated runs of data collection for Experiment 2 (Image adjustment task) using the decision criteria of*  BF_10_ or BF_01_ > 10  *and stopping at 250 participants.*

# S7. Summary tables

Table S1.

| Experiment 1 - Saturation | |
| --- | --- |
| Variable | Posterior mode and 95% HDI |
| Intercept [1 vs 8] | -4.64 [-4.94, -4.34] |
| Intercept [2 vs 8] | -3.65 [-3.94, -3.38] |
| Intercept [3 vs 8] | -2.83 [-3.13, -2.57] |
| Intercept [4 vs 8] | -2.18 [-2.43, -1.88] |
| Intercept [5 vs 8] | -1.53 [-1.76, -1.22] |
| Intercept [6 vs 8] | -0.54 [-0.80, -0.26] |
| Intercept [7 vs 8] | 0.81 [0.55, 1.09] |
| poly(Image manipulation Level,1) | 0.22 [0.13, 0.30] |
| poly(Image manipulation Level,2) | -0.32 [-0.39, -0.24] |
| CDS-State | -0.22 -[0.46, 0.01] |
| CDS-State:poly(Image manipulation Level,1) | -0.02 [-0.09, 0.06] |
| CDS-State:poly(Image manipulation Level,2) | 0.00 [-0.07, 0.07] |

| Experiment 1 - Contrast | |
| --- | --- |
| Variable | Posterior mode and 95% HDI |
| Intercept [1 vs 8] | -5.37 [-5.68, -5.05] |
| Intercept [2 vs 8] | -4.02 [-4.32, -3.72] |
| Intercept [3 vs 8] | -3.08 [-3.38, -2.79] |
| Intercept [4 vs 8] | -2.34 [-2.63, -2.04] |
| Intercept [5 vs 8] | -1.55 [-1.85, -1.27] |
| Intercept [6 vs 8] | -0.58 [-0.86, -0.28] |
| Intercept [7 vs 8] | 0.89 [0.61, 1.19] |
| poly(Image manipulation Level,1) | 0.55 [0.41, 0.72] |
| poly(Image manipulation Level,2) | -1.05 [-1.19, -0.90] |
| CDS-State | -0.20 [-0.47, 0.04] |
| CDS-State:poly(Image manipulation Level,1) | 0.02 [-0.11, 0.16] |
| CDS-State:poly(Image manipulation Level,2) | 0.09 [-0.05, 0.22] |

Table S2.

| Experiment 1 - Saturation: Extended CDS-State model | |
| --- | --- |
| Variable | Posterior mode and 95% HDI |
| CDS-State | -0.17 [-0.40, 0.09] |
| Image Category (Indoor vs Flower) | 0.62 [0.31, 0.89] |
| Image Category (Outdoor vs Flower) | 0.52 [0.22, 0.78] |
| Image Category (People vs Flower) | 1.07 [0.81, 1.38] |
| CDS-State:Image Category(Indoor vs Flower) | -0.05 [-0.14, 0.06] |
| CDS-State:Image Category(Outdoor vs Flower) | -0.12 [-0.22, -0.02] |
| CDS-State:Image Category(People vs Flower) | -0.12 [-0.21, -0.01] |

| Experiment 1 - Saturation: Extended CDS-Trait model | |
| --- | --- |
| Variable | Posterior mode and 95% HDI |
| CDS-Trait | -0.33 [-0.57, -0.10] |
| CDS-Trait:poly(Image manipulation Level,1) | -0.03 [-0.10, 0.04] |
| CDS-Trait:poly(Image manipulation Level,1) | -0.01 [-0.07, 0.06] |
| CDS-Trait:Image Category(Indoor vs Flower) | 0.16 [0.06, 0.26] |
| CDS-Trait:Image Category(Outdoor vs Flower) | 0.03 [-0.07, 0.13] |
| CDS-Trait:Image Category(People vs Flower) | 0.03 [-0.07, 0.13] |

| Experiment 1 - Contrast: Extended CDS-State model | |
| --- | --- |
| Variable | Posterior mode and 95% HDI |
| CDS-State | -0.23 [-0.47, 0.05] |
| Image Category (Indoor vs Flower) | 0.13 [-0.14, 0.38] |
| Image Category (Outdoor vs Flower) | 0.05 [-0.23, 0.30] |
| Image Category (People vs Flower) | 0.62 [0.36, 0.90] |
| CDS-State:Image Category(Indoor vs Flower) | 0.02 [-0.08, 0.11] |
| CDS-State:Image Category(Outdoor vs Flower) | 0.00 [-0.09, 0.10] |
| CDS-State:Image Category(People vs Flower) | -0.03 [-0.13, 0.07] |

| Experiment 1 - Contrast: Extended CDS-Trait model | |
| --- | --- |
| Variable | Posterior mode and 95% HDI |
| CDS-Trait | -0.21 [-0.46, 0.07] |
| CDS-Trait:poly(Image manipulation Level,1) | -0.01 [-0.16, 0.12] |
| CDS-Trait:poly(Image manipulation Level,1) | 0.09 [-0.04, 0.23] |
| CDS-Trait:Image Category(Indoor vs Flower) | -0.09 [-0.18, 0.02] |
| CDS-Trait:Image Category(Outdoor vs Flower) | -0.02 [-0.13, 0.07] |
| CDS-Trait:Image Category(People vs Flower) | -0.09 [-0.19, 0.00] |

Table S3.

| Experiment 2 - Saturation: Extended CDS-State model | |
| --- | --- |
| Variable | Posterior mode and 95% HDI |
| Intercept | 116.68 [110.22, 123.63] |
| CDS-State | -0.02 [-0.14, 0.11] |
| Image Category (Indoor vs Flower) | 0.06 [-1.99, 1.88] |
| Image Category (Outdoor vs Flower) | -0.24 [-2.04, 1.80] |
| Image Category (People vs Flower) | 0.59 [-1.49, 2.35] |
| CDS-State:Image Category(Indoor vs Flower) | 0.02 [-0.05, 0.08] |
| CDS-State:Image Category(Outdoor vs Flower) | 0.01 [-0.05, 0.07] |
| CDS-State:Image Category(People vs Flower) | -0.02 [-0.09, 0.04] |

| Experiment 2 - Saturation: Extended CDS-Trait model | |
| --- | --- |
| Variable | Posterior mode and 95% HDI |
| Intercept | 117.24 [110.23, 124.93] |
| CDS-Trait | -0.03 [-0.14, 0.09] |
| Image Category (Indoor vs Flower) | -0.14 [-1.98, 1.83] |
| Image Category (Outdoor vs Flower) | -0.05 [-2.02, 1.81] |
| Image Category (People vs Flower) | 0.46 [-1.53, 2.37] |
| CDS-Trait:Image Category(Indoor vs Flower) | 0.02 [-0.04, 0.07] |
| CDS-Trait:Image Category(Outdoor vs Flower) | 0.00 [-0.05, 0.05] |
| CDS-Trait:Image Category(People vs Flower) | -0.01 [-0.07, 0.05] |

| Experiment 2 - Contrast: Extended CDS-State model | |
| --- | --- |
| Variable | Posterior mode and 95% HDI |
| Intercept | 110.99 [105.34, 116.36] |
| CDS-State | -0.01 [-0.13, 0.13] |
| Image Category (Indoor vs Flower) | -0.28 [-2.10, 1.70] |
| Image Category (Outdoor vs Flower) | 0.10 [-1.71, 2.01] |
| Image Category (People vs Flower) | 0.24 [-1.69, 2.05] |
| CDS-State:Image Category(Indoor vs Flower) | -0.02 [-0.09, 0.05] |
| CDS-State:Image Category(Outdoor vs Flower) | -0.05 [-0.12, 0.02] |
| CDS-State:Image Category(People vs Flower) | -0.03 [-0.10, 0.03] |

| Experiment 2 - Contrast: Extended CDS-Trait model | |
| --- | --- |
| Variable | Posterior mode and 95% HDI |
| Intercept | 110.34 [104.46, 116.2] |
| CDS-Trait | 0.00 [-0.10, 0.10] |
| Image Category (Indoor vs Flower) | -0.34 [-2.24, 1.57] |
| Image Category (Outdoor vs Flower) | 0.00 [-1.79, 1.91] |
| Image Category (People vs Flower) | 0.30 [-1.73, 2.00] |
| CDS-Trait:Image Category(Indoor vs Flower) | 0.02 [-0.03, 0.08] |
| CDS-Trait:Image Category(Outdoor vs Flower) | -0.01 [-0.06, 0.04] |
| CDS-Trait:Image Category(People vs Flower) | -0.01 [-0.06, 0.04] |

#

###

# S6. Correlation between task performance (median response times per participants in Experiment 1 and 2, double-pass correlation per participants in Experiment 2) and CDS-scores

Model:

$median RT \sim CDSstate$

$$Pearson r \sim CDSstate$$

Priors:

$$\beta\sim Normal(0,1)$$

$$\sigma\sim Normal(0,1)$$

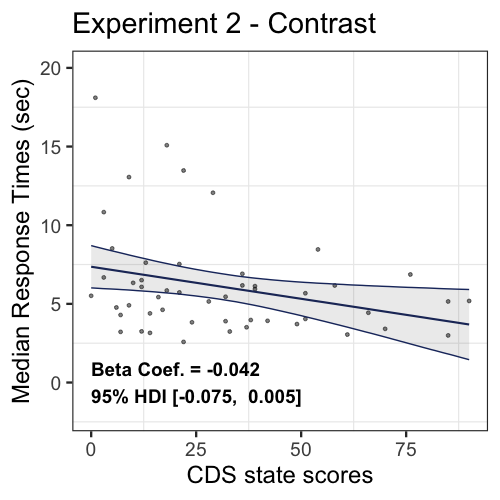

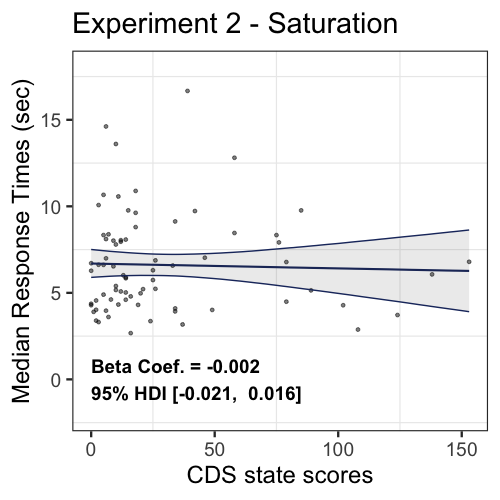

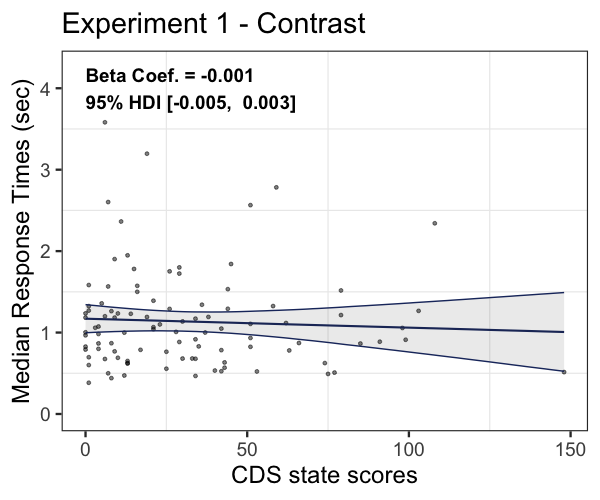
Figure S7. Median trial response times per participant in Experiment 1 & 2 and CDS state scores. Based on regression coefficients, we did not find increased response times associated with high CDS scores. Additionally, the double-pass correlations between responses in Experiment 2 were not correlated with CDS-state scores. (Saturation: β_CDS-State_= 0.000, 95% HDI [-0.001, 0.002]; Contrast: β_CDS-State_ = -0.001, 95% HDI [-0.003, 0.002])
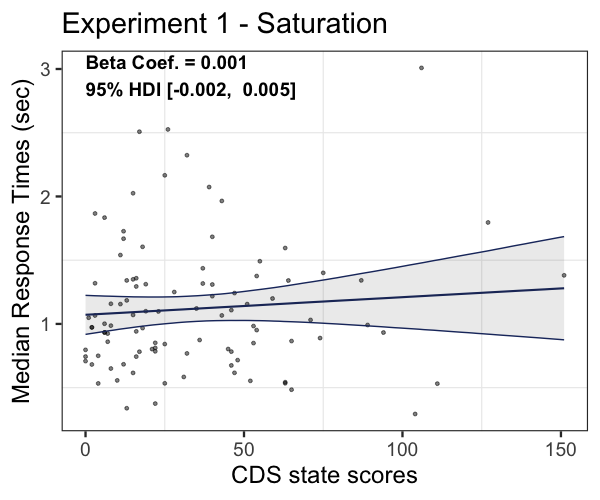
.

# 
