# Supplementary material for: Lifting the veil: probing altered visual perception in derealization
Source: Neurosci Conscious. 2025 Nov 23;2025(1):niaf045. doi: 10.1093/nc/niaf045 (PMC12640545; doi:10.1093/nc/niaf045)
Supplement: OPEN_SCIENCE_BADGE_APPLICATION_FORM_niaf045 [file open_science_badge_application_form_niaf045.docx]

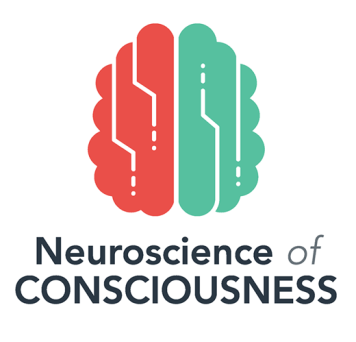
 **OPEN SCIENCE BADGE APPLICATION FORM**

**Open Data Badge**

Please provide the URL, DOI, or other permanent path for accessing the data in a public, open access repository.

I

https://osf.io/vefsd

Is there sufficient information for an independent researcher to reproduce the reported results? If no, explain.

Yes

**Open Materials Badge**

Please provide the URL, DOI, or other permanent path for accessing the materials in a public, open access repository.

I

https://osf.io/vefsd

Is there sufficient information for an independent researcher to reproduce the reported methodology? If no, explain.

yes

**Preregistered Badge**

Please provide the URL, DOI, or other permanent path to the registration (and, if applicable, the analysis plan) in a public, open access repository.

I

https://osf.io/vefsd

Was the plan preregistered prior to the examination of the data or observing the outcomes? If no, explain.

yes

Were there additional registrations for the study other than the one reported? If yes, provide links and explain.

no

For Preregistered and Analysis plan badge: were there any changes to the preregistered analysis plan for the primary confirmatory analysis? If yes, explain.

no

For Preregistered and Analysis plan badge: are all of the analyses described in the registered plan reported in the article? If no, explain.

yes
